# Consecutive Aromatic Residues Are Required for Improved Efficacy of β-Sheet Breakers

**DOI:** 10.3390/ijms23095247

**Published:** 2022-05-08

**Authors:** Adam Jarmuła, Monika Zubalska, Dariusz Stępkowski

**Affiliations:** 1 Laboratory of Bioinformatics, Nencki Institute of Experimental Biology, Pasteur 3 St., 02-093 Warsaw, Poland; 2 Faculty of Physics, University of Warsaw, Pasteur 5 St., 02-093 Warsaw, Poland; monikazubalska@gmail.com; 3 Laboratory of Molecular Basis of Cell Motility, Nencki Institute of Experimental Biology, Pasteur 3 St., 02-093 Warsaw, Poland; d.stepkowski@nencki.edu.pl

**Keywords:** β-sheet-breakers, dissolution of β-amyloid fibrils, molecular dynamics, peptides with consecutive aromatic residues

## Abstract

Alzheimer’s disease is a fatal neurodegenerative malady which up to very recently did not have approved therapy modifying its course. After controversial approval of aducanumab (monoclonal antibody clearing β-amyloid plaques) by FDA for use in very early stages of disease, possibly new avenue opened for the treatment of patients. In line with this approach is search for compounds blocking aggregation into amyloid oligomers subsequently forming fibrils or compounds helping in getting rid of plaques formed by β-amyloid fibrils. Here we present in silico work on 627 sixtapeptide β-sheet breakers (BSBs) containing consecutive three aromatic residues. Three of these BSBs caused dissociation of one or two β-amyloid chains from U-shaped β-amyloid protofibril model 2BEG after docking and subsequent molecular dynamics simulations. Thorough analysis of our results let us postulate that the first steps of binding these successful BSBs involve π–π interactions with stacked chains of F19 and later also with F20 (F3 and F4 in 2BEG model of protofibril). The consecutive location of aromatic residues in BSBs makes them more attractive for chains of stacked F3 and F4 within the 2BEG model. Spotted by us, BSBs may be prospective lead compounds for an anti-Alzheimer’s therapy.

## 1. Introduction

Alzheimer’s disease (AD) is characterized by accumulation in the brain of β-amyloid plaques and neurofibrillary tangles of hyper-phosphorylated tau protein [1]. The processes of forming these protein aggregates (amyloidogenesis) are extensively studied. Two consecutive phenylalanine (F) residues (19,20) in the sequence of β-amyloid 1-42 peptide are considered as one of the amyloidogenic centers. The pivotal role of aromatic stacking in the amyloidogenesis processes was proposed by Gazit [2,3]. Supposedly, interfering with this stacking would be a prospective way to destabilize β-amyloid fibrils or block the formation of fibrils. The precursor of all β-sheet breakers iAβ5 proposed by Soto et al. [4] contains such FF motif. The anti-amyloidogenic properties of iAβ5 were postulated as the ability to impede the formation of fibrils or to destabilize cross-β-structure by intercalation [4]. We have previously hypothesized that in the very beginning of the interaction of iAβ5 with mature fibril, internal π–π interactions within inhibitor molecule will improve anti-amyloidogenic potency of iAβ5 LPFFD (LeuProPhePheAsp) [5]. To demonstrate our point, one more consecutive F residue was added to the FF motif, constituting iAβ6 peptide LPFFFD (LeuProPhePhePheAsp). Its improved anti-amyloidogenic potency was confirmed in silico [5,6]. On the other hand, FF, FFF, FFFF homo-peptides form by themselves tubular or other forms of nano-structures depending on solvent conditions [7]. These structures are formed predominantly due to π–π stacking. Recently, in a very elegant experimental work, Koshti et al. [8] studied aggregated peptide FF nanostructure as the simplest model of amyloid-β fibril. Preformed FF nanofibers were treated with a variety of known non-peptide inhibitors of amyloidogenesis. All of these inhibitors contain aromatic moieties and all decomposed FF nanofibers through π–π interactions interfering with the π–π stacking of phenylalanines of FF nanofibers. Such stacking characterizes U-shaped structure of amyloid-β 17-42 (PDB code 2BEG) protofilament [9]. Both F19, located inside the structure, and F20, on the outer surface of the protofibril, form chains of π-stacked aromatic rings along the fibril axis. This prompted us to use F20 in chain C of 2BEG structure as a target for docking iAβ6 to this model of protofibril. Docking followed by molecular dynamics simulations (MDS) resulted in the dissociation of chain E from the protofibril for one docking pose and destabilization of the structure for many others [6]. In conclusion, interference of iAβ6 (rich in aromatic rings) with π-stacked rings of F20 leads to the destabilization of that particular protofibril model and eventually results in dissociation of one amyloid-β chain. It should be mentioned that fibrils of amyloid-β 1-42 are polymorphic in nature [10]. Besides U-shaped structures there exist also S-shaped [10] and LS-shaped [11] fibril conformers. The LS-shaped model presents F20 on the surface of protofibril, whereas F19 is buried inside the hydrophobic core of this structure. Summarizing, both amyloid-β phenylalanines, F19 and F20, are one of several targets for non-peptide and peptide inhibitors of amyloidogenesis, providing they contain aromatic residues. Indeed, inhibitors containing aromatic residues interfere with π-stacked along the fibril axis F19 and F20 by means of π–π interactions of their aromatic moieties with phenylalanine rings. There were attempts to optimize anti-amyloidogenic properties of peptide inhibitors belonging to the class of β-sheet breakers. Viet et al. [12] mutated tripeptides up to the number of 8000 variants. The best inhibitors were those containing two residues of tryptophan and one proline residue pointing to aromaticity as the main parameter of the potency of inhibitor. The aromatic additions to β-sheet breaker sequence were also studied by Kanchi and Dasmahapatra [13] using S shaped model of β-amyloid [10] and were found to improve binding of BSB molecule to this protofilament model. Shuaib et al. [14] studied 867 pentapetides variants of mutated iAβ5 and Jani et al. [15] studied 8 variants of 5 to 10-mer analogues of iAβ5. In both studies F20 and F19 were involved in binding of peptides to the protofibril model 2BEG. On the other hand, Dutta and Basu [16] mutated separately 13 residues in amyloid-β and studied the stability of the protofibril model 2BEG. They found important role of F19 and F20 in stabilizing the 2BEG structure by means of π–π stacking interactions. Surprisingly though, more pronounced role of F20 located on the surface of protofibril than F19 buried inside the hydrophobic core of the protofibril was observed. Besides the aromatic stacking also other stabilizing interactions such as salt bridges, inter-polypeptide hydrogen bonds, and hydrophobic contacts exist. In our previous hypothesis-driven work, F20 of chain C (in 2BEG structure) was the target of iAβ6. To clarify more the problem of which residue is the most important target for BSB in 2BEG protofibril model we performed free docking of 627 peptide analogues of iAβ6 followed by MDS of best 20 poses from docking. From these 20 chosen peptides, strong destabilization of 2BEG structure resulting in dissociation of either one amyloid-β chain or two chains was observed in two or one cases, respectively. Hence, our protocol let us find the most promising dissolving agents from the studied group of BSBs targeting the U-shaped model of β-amyloid fibrils.

## 2. Results and Discussion

### 2.1. Docking of 627 Sixtapeptides to the Aβ-Fibril-Model

**Figure 1:**
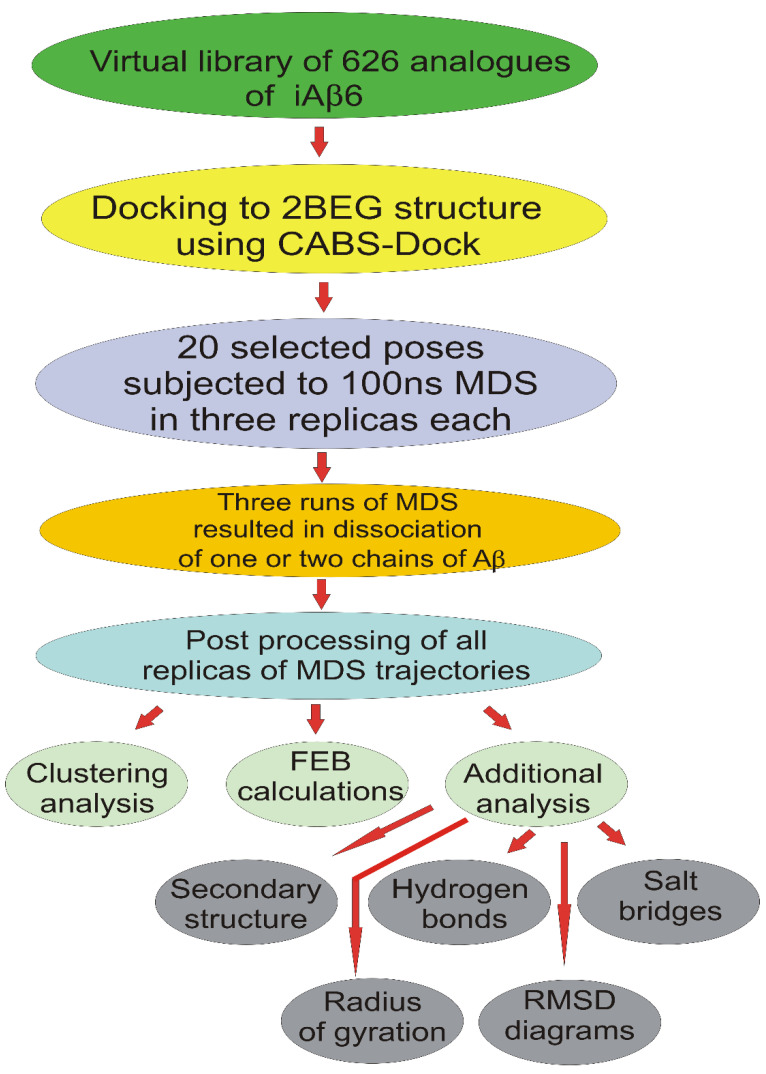
Schematic guide through the methodology.

In order to explore the initial binding of sixtapeptides from the 627-member library to the Aβ-fibril model, docking experiments have been performed with the CABS-Dock program. Each individual docking experiment resulted in the generation of 10 docking models. All obtained models have been compared and 20 best of them, including one model of LPFFFD as the control BSB peptide, selected for the next stage of the procedure, i.e., for molecular dynamics runs. The protocol used in our approach is presented in Figure 1.

### 2.2. Dissecting BSB Binding Conformations on the Basis of MDS Trajectories and Their Post-Processing

**Figure 2:**
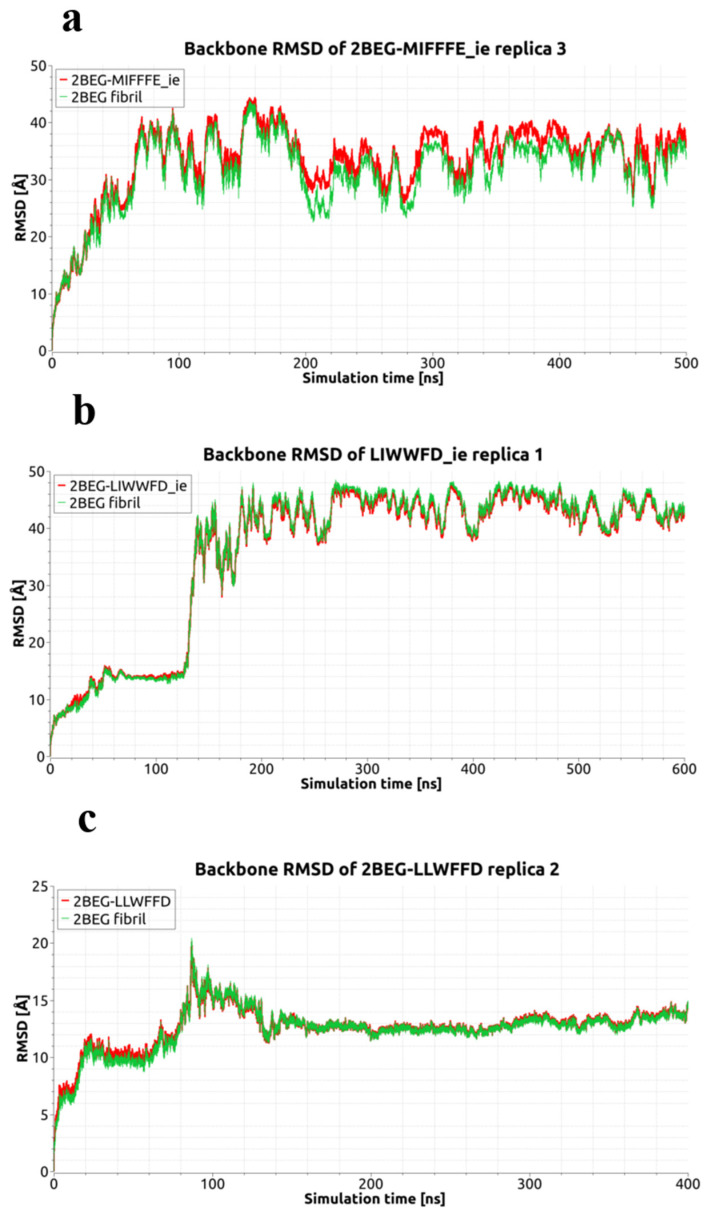
Backbone RMSD diagrams for 2BEG-MIFFFE_ie in MDS replica 3 (**a**), 2BEG-LIWWFD_ie in MDS replica 1 (**b**) and 2BEG-LLWFFD in MDS replica 2 (**c**). Red curves display RMSD for the 2BEG fibril together with ligand, while green curves for the fibril without ligand.

**Figure 3:**
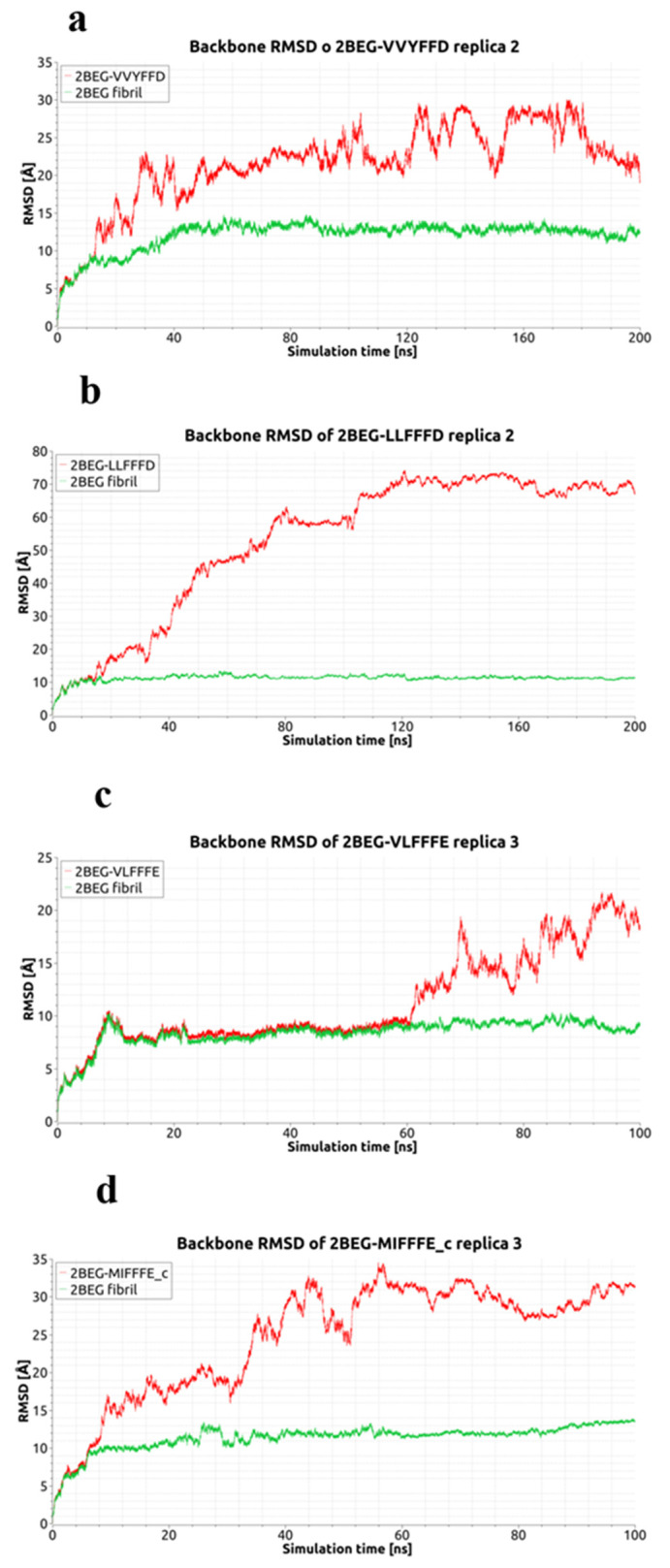
Backbone RMSD diagrams for 2BEG-VVYFFD in MDS replica 2 (**a**), 2BEG-LLFFFD in MDS replica 2 (**b**), 2BEG-VLFFFE in MDS replica 3 (**c**) and 2BEG-MIFFFE_c in MDS replica 3 (**d**). Red curves display RMSD for the 2BEG fibril together with ligand, while green curves for the fibril without ligand.

The MDS progress has been recorded in the RMSD diagrams (Figure 2 and Figure 3 and Supplementary Figure S1A–U) obtained both for whole systems (Aβ-fibril + ligand) as well as in some cases only for the receptor, i.e., Aβ-fibril molecule. The latter was applied for MD replicas where the dissociation of ligand had an occurrence, highlighting such an event by separate curve on the appropriate RMSD diagram (Figure 3). Majority of the RMSD diagrams spans the time period of 100 ns. However, in order to observe the dynamical/conformational effects in full, some of the MDS replicas have been extended from the basic 100 ns to 300–600 ns of duration. It was those cases where dissociation of the Aβ-fibril chains or close to dissociation events had been observed (Figure 2). The RMSD diagrams for both mentioned situations (dissociation of ligand or Aβ-fibril model chains) show that each dissociation changes the RMSD outlook by remarkably enlarging corresponding RMSD values (Figure 2 and Figure 3).

Clustering of the trajectories from MDS replicas of basic 100 ns runs resulted in strongly diversified numbers of clusters. Basically, depending on the clusters’ number, two groups of complexes can be distinguished: (1) the first one with 13 complexes spanning the range between 6 and 26 clusters and (2) the second one with remaining 8 complexes spanning the range between 42 and 154 clusters (Table 1). The first group consists of free Aβ-fibril model molecule as well as complexes, where ligand does too little to severely change the conformation of Aβ-fibril molecule. In the second group, however, ligands do much more to force the Aβ-fibril molecule to thoroughly penetrate available conformational space, resulting in much larger number of clusters and—in some instances—in the Aβ-fibril molecule being close to or even undergo dissociation of its outer chains from the original pentamer. In the cases of Aβ-fibril molecule dissociation, clustering for extended MD simulations has also been performed, both for all replicas together and for the particular replicas where dissociation took place. The results showed each time an increased number of clusters (Table 1).

The BFE for combined three replicas of basic MDS for each simulated system as well as their components are shown in Table 2.

The results clearly indicate less favorable free energies for the second group of complexes, characterized by larger numbers of clusters (*cf.* Table 1). The only exception in this group is the 2BEG-LIWWFD_ie system, for which the BFE amounts to −25.66 kcal/mol, thus being comparably favorable to the BFE in the first group of complexes, characterized by smaller numbers of clusters (*cf.* Table 1). The exception in the second group is the 2BEG-MIFFFE_c system, for which the BFE of −8.50 kcal/mol is the smallest in the entire pool of complexes, i.e., for the first and second group together. However, there seems to be no clear scheme of the contributions of the components of BFE to the global results. Depending on the particular trajectory, there are huge differences in the electrostatic energies and solvation free energies, whereas the van der Waals and non-polar free energies of solvation are roughly similar among the results. While the contributions from the van der Waals term are significant, non-polar solvation free energies are always small and seemingly non-decisive on the BFE energies. In Table 3, BFE energies with their components for particular basic MDS replicas where the Aβ-fibril molecule dissociation took place as well as together for two remaining replicas from the appropriate trajectories are presented.

These results emphasize the change in Aβ-fibril composition for the 2BEG-MIFFFE_ie complex by showing much less favorable BFE energy for the replica with dissociation. For the remaining two complexes, 2BEG-LLWFFD and 2BEG-LIWWFD_ie, the results are on the same level for replicas without and with dissociation, arguing for relatively rarer presence of dissociated Aβ-fibril states in the course of basic MDS. Indeed, the dissociation of chain A from 2BEG-LLWFFD complex is a short-living one and the dissociation of chain E from the 2BEG-LIWWFD_ie complex starts no sooner than at ca. 133 ns (see Figure 2A), so although this particular dissociation extends through all phases of extended MDS (up to 600 ns), it is yet not quite reflected in BFE computed after the first 200 ns of MDS.

Radii of gyration of the Aβ-fibril model molecule for combined replicas of basic trajectories are shown in Table 4.

The average values are similar among the complexes, spanning the range between 14.6 to 16.5 Å. The lowest radius of gyration of 14.6 Å is observed for the simulation of 2BEG molecule alone, not disturbed by ligands. The only two clear exceptions are the replicas with Aβ-fibril dissociation, where the average values grow up to 24.6 Å (2BEG-LIWWFD_ie complex) and 31.0 Å (2BEG-MIFFFE_ie complex). Due to relatively short time of dissociation of chain A from the Aβ-fibril molecule in the course of basic run of replica 2 of the 2BEG-LLWFFD complex, the corresponding radius of gyration from this replica is quite similar to the combined radii of gyration from all three replicas and from non-dissociating replicas 1+3.

The occupancies of the secondary structure (SS) of the Aβ-fibril model molecule are presented in Table 5.

The results from the combined replicas of basic MDS show that the occupancy of the most characteristic SS element in the Aβ-fibril molecule structure, the β-sheets (parallel + anti-parallel) is highest for the basic MDS of 2BEG alone (43%), whereas it lowers to different levels depending on the ligand. The lowest levels of 8% concern two complexes, where dissociations of Aβ-fibril model molecule in single replicas have been spotted, namely 2BEG-MIFFFE_ie and 2BEG-LIWWFD_ie. In the last complex with one dissociated replica, 2BEG-LLWFFD, the β-sheets occupancy is slightly higher, although still one of the lowest in the overall pool. In general, the assignments of SS correspond well with the results in Table 1 and Table 2, amounting to lower quantities of β-sheets for complexes with larger numbers of clusters and less favorable BFE and to higher ones for complexes with smaller numbers of clusters and more favorable BFE. Low and high SS occupancies of β-sheets are compensated by the occupancies of less regular structural elements, bends and mainly coils, to a level similar to all complexes, amounting to 89–98%. On the other hand, various types of helices are almost completely absent in the analyzed structures.

**Figure 4:**
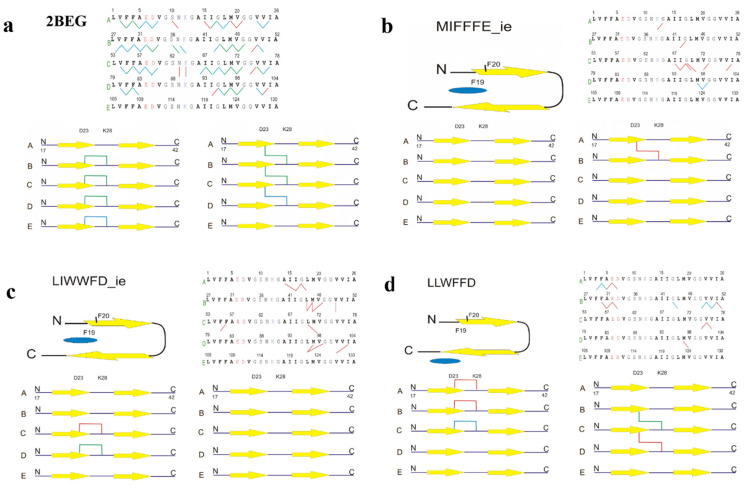
Topologies for structures after molecular docking, hydrogen bonds and intra-chain and inter-chain salt bridges occupancies from combined replicas of MDS: (**a**) for the fibril model 2BEG, (**b**) for 2BEG-MIFFFE_ie, (**c**) for 2BEG-LIWWFD_ie, and (**d**) for 2BEG-LLWFFD. The occupancies are color coded 30–50% red, 50–70% blue, and above 70% green.

Hydrogen bonds (HB) between chains of the Aβ-fibril model molecule are shown in Figure 4a–d and in Supplementary Figure S2A–Q in three thresholds: above 30, 50, and 70% of the occupancy during combined replicas of the basic MDS.

These results show the highest numbers of hydrogen bonds, both together from all thresholds (61) and from the finest 70% threshold (21), belong to the apo 2BEG structure, whereas all ligands disturb less or more the Aβ-fibril molecule, resulting in the reduction of some of, or even of the majority of, contacts. The sum of all HB present agrees well with the results in Table 1, Table 2 and Table 5, showing more HB contacts (in the range between 29–53) for complexes with smaller numbers of clusters, more favorable binding free energies, and higher occupancies of the β-sheets secondary structure, while less HB contacts (in the range between 6 and 15) for complexes with larger numbers of clusters, less favorable binding free energies, and lower occupancies of the β-sheets secondary structure. In particular, the complexes with one replica of dissociated Aβ-fibril molecule, 2BEG-MIFFFE_ie, 2BEG-LIWWFD_ie and 2BEG-LLWFFD, are described overall by only 10, 13, and 15 HB contacts, respectively, including null contacts with occupancies over 70%. Moreover, in some cases, the HB occupancies are clearly unsymmetrical, showing more soundly for outer chains AB or DE, like, for example, for the 2BEG apo structure and 2BEG-LIWFFD complex (more HB contacts connecting the A and B chains) or the 2BEG-PIFFWD (Supplementary Figure S2N) and 2BEG-GVFFFD (Supplementary Figure S2C) complexes (more HB contacts connecting the D and E chains), demonstrating unequal disruption of the fibril progressing either spontaneously, without ligand (apo 2BEG) or with the participation of ligand. The statistics of HB contacts between the Aβ-fibril model molecule and ligand in the basic MDS is shown in Supplementary Table S1. There are seldom HB contacts of occupancy exceeding 20% and only 2 contacts in 2BEG-LIWWFD_c exceed the 30% occupancy.

**Figure 5:**
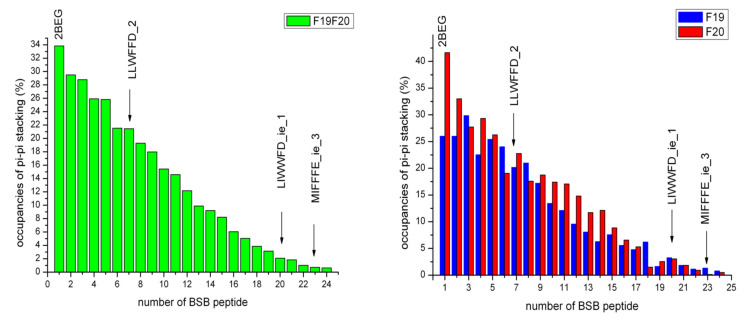
Statistics of stacking interactions between side chains of Phe residues of the Aβ-fibril molecule in the course of basic MDS. Left panel presents the summary of stacking occupancies of Phe19 and Phe20 together, whereas right panel shows them separately. The BSB peptides in complexes with 2BEG are numbered as follows: 1—2BEG apo, 2—PAFFWD, 3—AMYFFD, 4—LIWFFD, 5—LPFFFD, 6—GVFFFD, 7—LLWFFD_replica 2, 8—LIFWYD, 9—LLWFFD_replicas 1 + 3, 10—LVYWFD, 11—MVWFFD, 12—PIFFWD, 13—VVFFWD, 14—MIFFFE_c, 15—GPWFWD, 16—LIWWD_c, 17—LMWWFD, 18—VLFFFE, 19—VVYFFD, 20—LIWWFD_ie_replica 1, 21—LLFFFD, 22—LIWWD_ie_replicas 2 + 3, 23—MIFFFE_ie_replica 3, 24—MIFFFE_ie_replicas 1 + 2. Where no replica is indicated, all 3 replicas of MDS are included.

Statistics of stacking interactions (SI) between phenylalanine side chains of the Aβ-fibril molecule are shown in Figure 5.

Similarly to the case of HB contacts, these results indicate the highest number of SI for the 2BEG apo structure and relatively high numbers also for the 2BEG-PAFFWD, 2BEG-AMYFFD and generally other complexes with smaller number of clusters, i.e., those less perturbed by ligands. The lowest SI numbers apply to the complexes strongly disturbed by ligands, especially those with stable dissociations of Aβ-fibril chains, i.e., 2BEG-MIFFFE_ie and 2BEG_LIWFFD_ie. It should be noted that F20 π–π stacking is more resistant to the disturbing effect of BSBs, as we observe for majority of cases higher occupancies of stacking for F20 than for F19 (see Figure 5).

**Figure 6:**
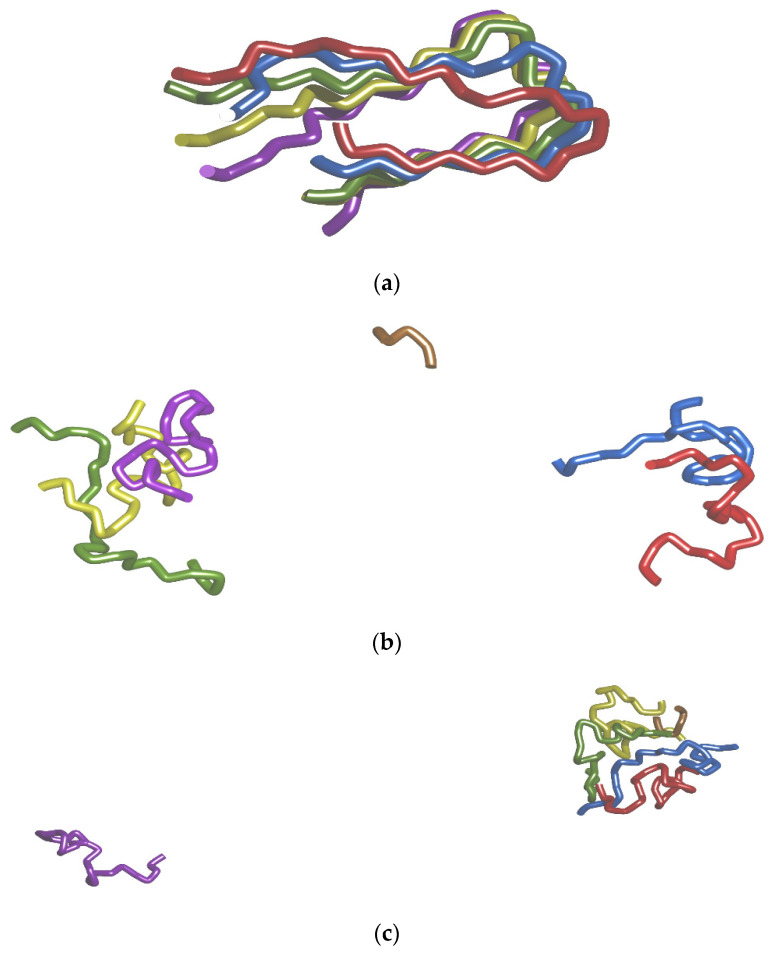

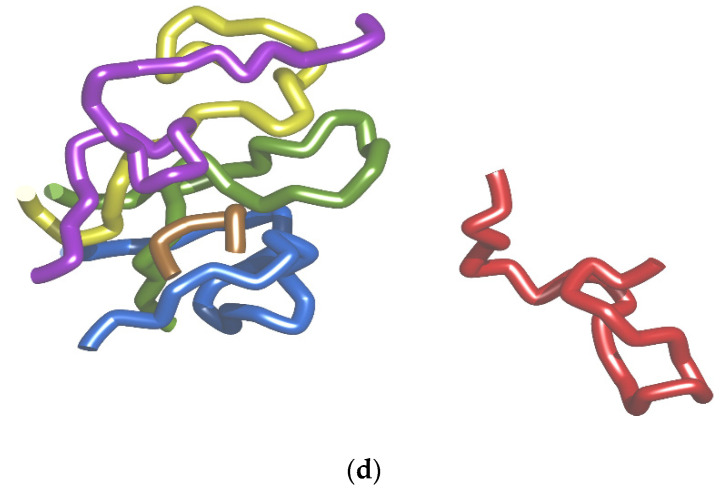
Representative structures from main/dissociating clusters from MDS for: (**a**)—2BEG fibril model (main cluster from combined replicas), (**b**)—2BEG-MIFFFE_ie complex (main cluster from replica 3), (**c**)—2BEG-LIWWFD_ie complex (third cluster from replica 1), (**d**)—2BEG-LLWFFD complex (34th cluster from replica 2). The chains are colored as follows: A—red, B—blue, C—green, D—yellow, E—purple. Ligand is colored brown.

Salt bridges (SB) between Asp 7 and Lys 12 in the same chain or Asp 7 in preceding and Lys 12 in succeeding chain are important components of the stability of the Aβ-fibril conformation modeled by the 2BEG structure. The statistics of global SB occurrences in both cases (for the same chain and for the two neighboring chains) in combined replicas of each complex undergoing basic MDS, including as well the 2BEG structure without ligands, are shown in Table 6 and the snapshot from combined replicas of MDS for 2BEG apo is shown in Figure 6a (figures with poses from main clusters or clusters with dissociating ligands can be found in Supplementary Figures S3A–F, S3H–J, S3L–N, S3P or S3G (3rd cluster of 2BEG-LLFFFD), S3K (3rd cluster of 2BEG-MIFFFE_c), S3O (4th cluster of 2BEG-VLFFFE), S3Q (3rd cluster of 2BEG-VVYFFD), respectively.

The hierarchies observed in Table 6 remember hierarchies present in Table 1, Table 2, Table 4 and Table 5, although there are some differences, including a few 2BEG complexes advancing to the first 13-member group and a few other landing in the second 8-member group (for comparison, see the clustering results in Table 1). However, the global SB results do not show the whole picture, neglecting the diversification of the SB occurrences among the chains of the Aβ-fibril model molecule. The latter can be conceived from panels C and D the presence of salt bridges in Figure 4 and Supplementary Figure S2A–Q. For example, the 8-th out of 21 result of the global SB occurrence between preceding/succeeding chains of the 2BEG-LLWFFD complex does not acknowledge relatively low occurrences of the salt bridges between the A-B and D-E chains of Aβ-fibril, an important thing considering the occurrence of a short-living dissociation of chain A in the second replica of MDS.

### 2.3. Dissociations of Selected Chains of Aβ-fFbril in Three MD Simulations

#### 2.3.1. MIFFFE_ie–Chains A and B

Before the dissociation process starts, chains A and B are thoroughly stabilized by many contacts to each other as well as to the neighboring chain C and to the ligand. The MIFFFE_ie ligand is placed inside the β-amyloid structure, being encircled by chain B and partly blocking the access to it from chain C and the rest of the β-amyloid molecule. However, only after some rearrangement of the ligand placement, the dissociation of chains B and A could happen. Indeed, such a rearrangement takes place in the form of the ligand moving toward chain C and acquiring additional contacts with it, while little by little losing its contacts with chain B. This rearrangement initially places the ligand between the N-terminal parts of chains B and C, while allows for the C-terminal and middle parts of chains B and C to ride off and step by step “throw off” the contacts between them. This way, the ligand gradually becomes a chock dividing the β-amyloid molecule into two parts, composed of chains A and B (first part) and C, D, and E (second part). Characteristically, chains C, D, and E place themselves in a roughly parallel order, which strengthens their inner coordination but makes less room for the contacts with chains B and A, staying partly masked by the ligand. Besides, at this time chains A and B considerably extend, which additionally reduces their area of contact with chain C, remaining, similarly to chains D and E, more coiled. Eventually, chain A moves away from the β-amyloid molecule, entailing chain B, which for a short time remains coordinated on the N-terminal part by chain C and the ligand. It leads to a strongly bent conformation, with a half of chain B on the side of N-terminus rising up vertically toward chain C and the second half on the side of C-terminus placed perpendicularly to it by following along chain A. At last, chains A and B free themselves from the coordination received from chain C and ligand and dissociate from the rest of β-amyloid molecule. From chain B, the most persistent interactions before the dissociation involve: (a) to chain C: Val 28 (hydrophobic contact to Phe 55), Phe 29 (hydrophobic contact to Leu 53), Phe 30 (H-bonds with Phe 55, Phe 56, Ala 57, hydrophobic contact to Ala 57), Ala 31 (hydrophobic contact to Ala 57), Asp 33 (H-bonds with Asn63, Lys 64), Gly 35 (H-bonds with Asn 63, Lys 64), Ser 36 (H-bond with Asn 63), Lys 38 (hydrophobic contacts to Lys 64, Ile 68), Ile 41 (hydrophobic contact to Ala 66), Leu 44 (hydrophobic contacts to Ile 67, Ile 68), (b) to the ligand: Leu 27 (hydrophobic contact to Met 131), Val 28 (hydrophobic contact to Met 131), Gly 47 (H-bond with Phe 135). Snapshot from replica 3 of MDS showing the dissociated Aβ-fibril molecule is shown in Figure 6b. Film with the dissociation progress, named Supplementary Movie S1, can be found in Supplementary Materials.

#### 2.3.2. LIWWFD_ie–Chain E

Stabilization of chain E directly before the dissociation event, involves the rests of Val 106, Ala 109, Glu 110, Asp 111, Ser 114, Asn 115, Leu 122, Val 124, Gly 125, Gly 126, and Val 127 interacting with amino-acids from chain D and to some extent with ligand. The Phe 107 and Phe 108 aromatic rings are stacked together, but being placed considerably outwards of the β-amyloid molecule, do not participate in stabilizing interactions with other chains. First, the ligand is pulled away and loses its contact with chain E, for which it gains full compensation as a result of more thorough coordination from chains B, D, and C. Next, chain E starts to migrate away and gradually losing its contacts with chain D. It starts from the C-terminal parts of the chain (except for hydrophobic interactions of Val 106 with Phe 82, holding on for a relatively long time, and of Phe 108 with Phe 82, appearing for a rather short time after a flip of the side chain of Phe 108 toward chain D). The resultant configuration of chain E, being coiled in a such way that its N- and C-termini do not border chain D, causes the middle parts of chain E to retain the longest contact time with chain D, resulting in the most long-living interactions between those chains on the threshold of dissociation belonging to the amino-acids of Ser 114, Asn 115 and, from time to time, Lys 116. Finally, first the C-terminus of chain E drifts toward, while the rest of chain E away from, chain D, and afterwards the C-terminus drifts again away from, while the middle parts of chain E come closer, to chain D, making room for the final interactions keeping chain E connected to the rest of the β-amyloid molecule, belonging first to the tercet of valines, 124, 127, and 128, duet of glycines, 125 and 126 and Ala 130, and afterwards to Ser 114, Asn 115, Gly 117, Ile 119 and Met 123 (all to chain D), as well as Leu 122 (to Leu 131 from the ligand). Eventually, the full dissociation of chain E occurs. Snapshot from replica 1 of MDS showing the dissociated Aβ-fibril molecule is shown in Figure 6c. Film with the dissociation progress, named Supplementary Movie S2, can be found in Supplementary Materials.

#### 2.3.3. LLWFFD–Chain A

Just before the start of dissociation, chain A remains thoroughly stabilized on both ends, with N-terminal contacts (both H-bonds and hydrophobic interactions) to a duet of phenylalanines, 3 and 4, and C-terminal extensive hydrophobic interactions between Val 20 and Val 23 of chain A and corresponding valine residues from the neighboring chain B and—in a single instance—chain C. The erosion of stabilizing contacts starts from the N-terminal residues of chain A moving away from its partner residues in chain B (first Phe 4, next Phe 3). It progresses such that the N-terminal together with the middle chain residues camber to the outside, while the C-terminal parts remain “glued” to their counterparts in chain B and sporadically chain C. At this time, the ligand occupies a place on the other side of chain B, from where it makes three hydrophobic contacts to this chain, as well as many other contacts to chains C, D, and especially E. Soon its contacts to chain B and then chain C are lost, which makes both those chains somewhat more free to move. At last, the C-terminal part of chain A loses its contacts to chains B and C by drifting away to the outside. The whole chain A cambers in such a way that the N-terminal part lands again in front of chain B and the C-terminus of chain C. It is the moment where chain A retains two contacts: an electrostatic attractive interaction of Glu 6 with Lys 38 from chain B and hydrophobic connection of Phe 4 with Ala 78 from chain C. After a short while, C-terminus drifts farther away, entailing the whole chain A. Two mentioned contacts disappear and it is the hydrophobic contact of the pendant side chain of Phe 3 with Ile 41 from chain B as the last instance of keeping the molecule of β-amyloid together, i.e., before the ultimate dissociation of chain A. Snapshot from replica 2 of MDS showing the dissociated Aβ-fibril molecule is shown in Figure 6d. Film with the dissociation progress, named Supplementary Movie S3, can be found in Supplementary Materials.

### 2.4. The Variety of BSB Targets

In our previous works [5,6] we have suggested Phe 20 of β-amyloid peptide forming 2BEG structure as an effective target of β-sheet breakers (BSB). The aromatic ring of Phe 20 is located on the outer surface of 2BEG U-shaped structure of β-amyloid 17–42 pentamer. Such location of Phe 20 in succeeding chains results in electron π coupling between Phe 20 aromatic rings along the fibril pentamer, which facilitates an attack of BSB rich in phenylalanines rings such as iAβ6. This attack involves internal π–π coupling within the inhibitor’s molecule. In the present studies we used an alternative approach letting the inhibitor molecule to semi-blind dock to the 2BEG structure receptor. From 627 studied different inhibitor peptides a selection of 20 was chosen for further analysis according to the criteria described in Methods. The topologies of the docking results are presented in Figure 4 and Supplementary Figure S2A–Q). From twenty configurations one presented the inhibitor molecule directed toward Phe20 (Supplementary Figure S2B (GPWFWD)), two on the C terminal part (Figure 4d (LLWFFD) and Supplementary Figure S2Q (VVYFFD)), and the rest inside the 2BEG structure, one deep inside (Supplementary Figure S2L (MVWFFD)), and remaining attacking the hydrophobic core including Phe 19. We conclude that the hydrophobic core with Phe 19 is the main target of β-sheet breakers studied in this paper.

Among the twenty peptides, two were most effective in demolition of the 2BEG structure during MD simulations, resulting finally in the dissociation of two β-amyloid chains (MIFFFE_ie) or one chain (LIWWFD_ie). Yet another dissociation, though an unstable and shortly present one, was also evidenced for LLWFFD. The extent of the disturbing effect can be judged visually on Figure 4 presenting network of hydrogen bonds stabilizing cross β-structure of 2BEG, and both internal and external salt bridges. In addition, in the stable cases of β-amyloid chains dissociation, π–π coupling of both Phe20 and Phe 19 side chains was strongly disturbed (see Figure 5).

### 2.5. Role of Consecutive Aromatic Residues in β-Sheet Breakers

All 627 peptides studied by us contain three consecutive aromatic residues, either F, W, or Y, preceded by two random hydrophobic residues and D or E as the C-terminal residue. We postulate that such an arrangement of consecutive aromatic residues facilitates internal π–π stacking within inhibitor molecule [5]. Such π–π stacking will cause shift of π-electrons making the aromatic rings of inhibitor molecule more attractive for their hypothetical target of Phe 19 or Phe 20 of β-amyloid. We postulate that the very first events of docking the inhibitor peptides involve aromatic π–π interactions. For the two of the three most effective peptides (MIFFFE_ie and LIWFFD_ie), the 2BEG π–π stacking along the fibril axis of both Phe20 and Phe19 was thoroughly disturbed during MDS (see Figure 5). Among this duo, the MIFFFE_ie ligand attacking the hydrophobic core of β-amyloid appears as the more effective one. It causes dissociation of two β-amyloid chains and results in most disturbed hydrogen bonds’ and stacking interactions’ networks and broken both intra- and inter-chain salt bridges. This observation allows us to conclude that the hydrophobic core containing Phe19 is the most kinetically effective target of β-sheet breakers with consecutive phenylalanines. The two bulky hydrophobic residues at the N-terminus of the studied iAβ6s appear also as necessary requirement for effective β-sheet breaker. In our studies the presence of Pro at the N-terminus is not so important. Proline is known as β-sheet breaker amino acid and was used for this purpose in the design of iAβ5 inhibitor [4]. This strategy was also employed in a paper by Kanchi and Dasmahapatra [17], who observed increased disrupting potency of KLVFFPPPPP by comparison with peptide KLVFF. However, they used S-shaped structure of β-amyloid protofibril. We postulate that the mechanism for dissolution of U-shaped β-amyloid fibrils is different and includes π–π interactions between inhibitor and phenylalanines of β-amyloid, which can be facilitated further by internal π–π coupling within inhibitor molecule. Besides the U-shaped 2BEG structure of β-amyloid fibril there are S-shaped and LS-shaped structures as well as other proteins forming amyloid fibrils. Elucidation whether the mechanism presented in this paper is a general one, applicable to all amyloid fibrils as well as to prevent formation of oligomers [18], will need further studies.

## 3. Methods

### 3.1. Generation of Virtual Library of 627 Sixtapeptides as Potential Inhibitors of β-Amyloidogenesis

Based on the conception introduced in [14], the sixtapeptide’s virtual library has been designed starting from the reference LPFFFD peptide [5,6]. Peptides have been generated by introducing one, two or, at the most, three mutations at different positions of LPFFFD using the SCWRL4 program [19]. Mutations were designed according to the following scheme acquired from Shuaib et al. [14]: L mutated with A, G, I, M, P or V, P with A, G, I, L, M or V, F with W or Y and D with E. This procedure resulted, together with the reference peptide LPFFFD, in a library of 627 sixtapeptides.

### 3.2. Molecular Docking of Sixtapeptides from the 627-Member Library to the Model of Aβ-Fibril

As a model of Aβ-fibril, the structure 2BEG from Protein Data Bank [9] has been used. It is an NMR structure consisting of five parallel chains forming the shape of horizontally mounted letter U. Among the pool of Aβ-fibril models available in PDB, the 2BEG structure has been selected due to its simplicity and compact form. It is worth to notice that on account of polymorphic character of Aβ-fibrils [10,20], the 2BEG structure represents one of the possible topological states of the fibril.

Molecular docking was performed with the CABS-Dock program [21] available online on the server in the Laboratory of Theory of Biopolymers at the Chemistry Faculty of Warsaw University. In docking the standard values of parameters, the number of the Monte Carlo simulation cycles (50) and the automatic prediction of the secondary structure of docked peptides by using the PSIPRED program [22] have been used. Probable binding sites of ligands on the surface of Aβ-fibril have been selected according to the indications of the machine learning method for the prediction of ligand binding sites on the surface of a selected protein receptor, P2Rank [23]. The mentioned binding sites on the surface of Aβ-fibril (residues 17–19 in chain A, 17, 19 and 40 in chain B, 17, 19, 40 and 42 in chains C and D, and 17, 19 and 38–40 in chain E) as well as the whole ligands were treated in docking as fully flexible. Finally, 20 best poses from docking have been selected for next steps of the procedure, consisting of molecular dynamics simulations and their subsequent analyses. The best poses have been selected according to the following criteria: (1) The largest number of molecular contacts between the Aβ-fibril molecule and ligand; (2) The most favorable interaction energy between the Aβ-fibril molecule and ligand, and (3) The most favorable overall energy (Aβ-fibril molecule + ligand).

### 3.3. Molecular Dynamics Simulations of Selected Poses from Docking

A total of 20 selected poses from the results of docking of iAβ6 and analogous ligands to the Aβ-fibril model and for the sake of control the Aβ-fibril molecule without ligands have been treated with molecular dynamics simulations (MDS) in order to describe the key interactions between the fibril and ligand as well as to decipher possible mechanisms of destabilization of the fibril by ligands. To effectively enhance the conformational space visited during MDS, each of the selected poses was simulated in three independent replicas of 100 ns-long MDS, each replica started with different distribution of atoms’ initial velocities. MDS have been carried out on tcr server in the Centre of New Technologies of Warsaw University using the pmemd program from Amber 18 [24] in the version for graphical processors. The fibril-ligand systems have been parametrized for simulations by employing the atomic types and charges from the ff14SB force-field [25]. Protonation states of all amino-acids have been assigned according to physiological pH. Monovalent sodium ions (counterions) have been added to warrant a full neutralization of the systems. The systems have been solvated with truncated octahedron boxes filled with TIP3P water molecules [26] extended up to 12 Å from the protein surface. Periodic boundary conditions have been used in all molecular dynamics (MD) runs.

The following simulation protocol has been used: solvated systems were optimized in two-step minimization procedure (first solvent and counterions, later the whole system) to relieve steric clashes and let the system to adopt a smooth initial conformation. During both minimization steps first the steepest descent and then conjugate gradient algorithms were used in proportion 10% to 90%, respectively. Next, the systems were gradually heated to target temperature of 300 K in three 7ps intervals (0 to 100 K, 100 K to 200 K and 200 K to 300 K). Equilibrations for 99 ps in the temperature of 300 K, followed by 3 replicas of data collection runs, each for 100 ns, finished the basic MDS phase. The isothermal-isobaric NpT ensemble (conserved number of atoms N, pressure p and temperature T) was used throughout the simulations. The temperature and pressure were maintained at 300 K and 1 atm, respectively, using the isotropic position scaling and the Berendsen barostat with weak-coupling algorithm [27], respectively, with both coupling times of 1 ps. A 1fs integration time step was used throughout the simulations. The SHAKE algorithm [28] was used to constrain all bonds involving hydrogens. The long-range Lennard-Jones and electrostatic interactions were calculated with particle-mesh Ewald (PME) method [29,30]. In the course of data collection MDS, atomic coordinates were saved every 2 ps, resulting in the trajectory record consisting of 50,000 structures for each 100 ns-long replica of MD run. Selected MDS have been extended to 300–600 ns of duration.

### 3.4. Post-Processing of the Trajectories from MDS

#### 3.4.1. Clustering Analysis Protocol

Combined clustering analyses of three replicas of MD runs for each simulated system have been performed with the cpptraj module [31] of AMBER 18 [24]. Successive frames from three replicas have been RMSD-fitted to the initial frame of replica 1 in order to eliminate translational and rotational motions from the analyses. The structures have been clustered with the hierarchical average-linkage algorithm using RMSD as similarity metric [32]. In order to obtain the most stable clustering results and especially to exclude high-frequency side chain motions, clustering has been constricted to the Cα atoms. Various clustering schemes have been tried including the minimum distance between clusters exceeding either 4 or 4.5 or 5 Å, with the 4.5 Å clustering scheme eventually selected as giving the most stable results. Preferred clusters’ numbers have been chosen based on the following criteria: Davies-Bouldin (DBI) index [33], pseudo F-statistic (pSF) [34], and SSR/SST parameter. The DBI index aims to identify clusters that are compact and well-separated. Lower DBI values are indicative of better clustering. The pSF statistic is a measure of the “tightness” of clusters; higher values indicate better clustering. At last, the SSR/SST parameter informs on the amount of variability explained in clustering results (the parameter is equivalent to the determination coefficient R^2^ [35])—its higher values reflect better clusterization. The same protocol was applied to extended MDS.

#### 3.4.2. Free Energy of Binding Computations

The binding free energies (BFE) of the selected sixtapeptides to the Aβ-fibril model have been calculated with the Molecular Mechanics-Generalized Born Surface Area (MM-GBSA) approach [36]. The binding free energy can be computed according to the formula: ΔG_bind_ = ΔE_mm_ + ΔG_solv_ - −TΔS_solute_, where ΔE_mm_, ΔG_solv_, and TΔS_solute_ are the molecular mechanics energy, solvation free energy, and entropy, respectively, contributions to Δ G_bind_. ΔE_mm_ can be expressed as the sum of changes in electrostatic (ΔE_ele_)_,_ van der Waals (ΔE_vdW_), and internal (ΔE_int_) energies in the gas-phase (ΔE_mm_ = ΔE_ele_ + ΔE_vdW_ + Δ E_int_), whereas ΔG_solv_ as the sum of changes in polar (ΔG_GB_) and non-polar (ΔG_np_) free energies of solvation (ΔG_solv_ = ΔG_GB_ + Δ G_np_). After discarding water and counterion molecules, ΔE_mm_ energies for complex, receptor, and ligand have been computed using the dielectric constant of 1 and an infinite cutoff for all interactions. ΔG_GB_ free energies for complex, receptor, and ligand have been computed using a continuum representation of the solvent and the modified generalized Born solvation model II [37]. Dielectric constants of 1 and 78.5 have been assigned to the solute (receptor+ligand) and solvent, respectively. Non-polar free energies of solvation for the complex, receptor, and ligand have been evaluated as contributions based on the solvent accessible surface area (SASA) computed with the LCPO method [38]. The solute entropy contributions to the binding free energy have not been calculated due to known difficulties in their accurate assessment [39]. All MM-GBSA computations have been performed for combined three replicas of MDS of each investigated system with the tools from Amber 18 [24]: the MMPBSA.py script [40] and SANDER program. Finally, the binding free energies (without entropy contributions) in this work are calculated as: Δ*G*_bind_ = Δ*E_ele_* + Δ*E_vdW_ +* Δ*G*_GB_ + Δ*G*_np_. Δ*E*_int_ have been not included, because their contributions sum up to 0 when calculated as herein, i.e., using the single trajectory approach. The same protocol was applied to extended MDS.

#### 3.4.3. Additional Analyses of MDS Trajectories

Available trajectories have been also analyzed in other aspects. All mentioned herein analyses have been carried out using the sum of conformational spaces from all replicas of a particular system. Hydrogen bonds between individual chains in Aβ-fibril model and between the fibril and ligand have been computed according to the following geometric criteria: the donor-acceptor distance not exceeding 3.2 Å and the donor-hydrogen-acceptor angle equal or exceeding 120°. Stacking interactions with cutoff distances of 5.5 Å between Phe residues in neighboring chains of Aβ-fibril model have also been evaluated. The occurrence of inter-chain salt bridges at the bending region of the fibril model stabilizes its conformation. The criterion for the presence of salt bridges in the trajectory was the distances between atoms of CG in Asp at position 7 and NZ in Lys at position 12, either in the same chains or in the two neighboring chains of the fibril model, not exceeding 4.5 Å. The mass-weighted radii of gyration for the Aβ-fibril model have been calculated in order to estimate the compactness of the fibril structures in the conformational space of MD runs. The occupancies of the secondary structure of the Aβ-fibril model and in particular of the β-sheets strapping together the fibril chains, have also been calculated.

RMSD diagrams for the main chains of the investigated complexes have been calculated for each MD replica.

## 4. Conclusions

The protofibril model structure 2BEG (U-shaped) is stabilized by a networks of interactions both within and between chains of β-amyloid. Among them the π–π coupling of the aromatic rings of F19s inside the 2BEG structure and F20s on the outer surface are promising targets for the attack of BSBs leading to dissolution of protofibril. According to our results, addition of one consecutive aromatic residue to the iAβ5 sequence leads to improvement of dissolving potency of resulting iAβ6 analogues. The most efficient BSB peptide MIFFFE docks inside the 2BEG structure, being initially coordinated by π–π stacking with F19. MDS of this molecular assembly leads to full dissociation of two β-amyloid chains from the protofibril structure. From the other BSBs that are able to dissociate the protofibril model, one attacks 2BEG structure similarly to MIFFFE, the other one docks to C-termini of β-amyloid chains on the outer side of the protofibril. However, the later one is least effective. We conclude that the most effective way to dissociate the U-shaped model of β-amyloid protofibril is to initially dock to and break the π–π coupling of F19 aromatic rings. Whether our BSBs will be effective in case of other than 2BEG model conformers of β-amyloid fibrils or other proteins forming amyloid fibrils remains an open question and needs further studies.

## Figures and Tables

**Table 1:** Cluster’s numbers during basic (100 ns) and, in cases of dissociation of the Aβ-fibril model molecule, extended MDS. ds stands for replicas with dissociation of Aβ-fibril molecule.

2BEG Complex with	No. of Clusters 100 ns	No. of Clusters 100 ns ds	No. of Clusters Extended MDS	No. of Clusters Extended MDS ds
LIWFFD	6			
PAFFWD	7			
apo 2BEG	10			
LVYWFD	11			
VLFFFE	11			
PIFFWD	13			
AMYFFD	15			
MIFFFE_c	16			
MVWFFD	18			
GVFFFD	20			
LIFWYD	21			
LIWWFD_c	23			
LPFFFD	26			
LLWFFD	42	30	82	50
GPWFWD	46			
VVFFWD	52			
LMWWFD	60			
LLFFFD	61			
VVYFFD	70			
MIFFFE_ie	154	107	488	380
LIWWFD_ie	186 *	127 *	371	264

* MDS duration of 200 ns.

**Table 2:** Binding free energies [kcal/mol] and their contributions for 20 complexes undergoing basic MDS. Values in parentheses are standard deviations.

Complex of 2BEG with	Δ*E*_ele_	Δ*E*_vdW_	Δ*G*_GB_	Δ*G*_np_	Δ*G*_solv_	Δ*G*_bind_
LIWFFD	−60.9	−51.8	90.4	−7.1	83.4	−29.4 (8.2)
PAFFWD	−17.0	−43.6	38.3	−5.6	32.7	−27.8 (9.4)
LVYWFD	−13.1	−39.3	40.9	−5.4	35.5	−16.9 (8.3)
VLFFFE	2.3	−36.6	19.7	−5.2	14.5	−19.8 (13.7)
PIFFWD	−12.2	−42.2	36.2	−5.8	30.4	−24.0 (9.3)
AMYFFD	−211.3	−40.4	229.9	−6.5	223.4	−28.3 (9.4)
MIFFFE_c	−25.0	−19.9	39.4	−3.0	36.3	−8.5 (7.9)
MVWFFD	73.6	−53.5	−42.2	−7.0	−49.2	−29.1 (8.9)
GVFFFD	−51.9	−37.5	72.3	−5.4	66.9	−22.5 (9.3)
LIFWYD	−42.7	−33.2	60.7	−4.6	56.1	−19.8 (7.8)
LIWWFD_c	−63.8	−50.2	85.5	−6.8	78.7	−35.3 (13.1)
LPFFFD	34.4	−33.6	−13.3	−4.5	−17.8	−17.0 (6.8)
LLWFFD	29.5	−38.9	−5.8	−5.2	−11.0	−20.3 (10.1)
GPWFWD	−17.5	−26.4	37.4	−3.9	33.5	−10.5 (9.5)
VVFFWD	27.6	−31.3	−4.2	−4.2	−8.5	−12.2 (11.3)
LIWWFD_ie	−92.2	−47.2	120.3	−6.6	113.7	−25.7 (9.4)
LMWWFD	−10.4	−27.2	28.8	−3.8	25.0	−12.6 (7.8)
LLFFFD	−23.7	−17.2	37.1	−2.5	34.6	−6.3 (7.3)
VVYFFD	26.3	−20.5	−14.0	−3.1	−17.1	−11.2 (10.9)
MIFFFE_ie	−6.4	−26.9	26.1	−4.0	22.1	−11.1 (10.3)

**Table 3:** Binding free energies [kcal/mol] and their contributions for basic MDS replicas of three complexes where dissociation of Aβ-fibril model molecule took place. Values in parentheses are standard deviations.

Complex of 2BEG with	Δ*E*_ele_	Δ*E*_vdW_	Δ*G*_GB_	Δ*G*_np_	Δ*G*_solv_	Δ*G*_bind_
LLWFFD rep. 2	33.4	−36.8	−12.1	−5.1	−17.2	−20.6 (8.3)
LLWFFD rep. 1 + rep. 3	27.6	−39.9	−2.8	−5.2	−8.0	−20.2 (10.8)
LIWWFD_ie rep. 1 *	−101.9	−45.2	125.0	−6.3	118.8	−28.3 (9.8)
LIWWFD_ie rep. 2 + rep. 3 *	−105.8	−48.3	132.2	−7.0	125.3	−28.8 (9.5)
MIFFFE_ie rep. 3	12.9	−10.1	−4.2	−1.5	−5.7	−3.0 (5.7)
MIFFFE_ie rep. 1 + rep. 2	−15.7	−35.2	41.0	−5.2	35.8	−15.1 (9.6)

* MDS duration of 200 ns.

**Table 4:** Average radii of gyration [Å] after various times of MDS for the 2BEG-ligand complexes. All stands for full three replicas, ds for replica with dissociation of the Aβ-fibril molecule, and nds for the remaining two replicas with no Aβ-fibril dissociation events.

2BEG Complex with	100 ns All	100 ns ds	100 ns nds	200 ns All	200 ns ds	200 ns nds	300 ns All	400 ns All	400 ns ds	400 ns nds	500 ns All	500 ns ds	500 ns nds	600 ns All	600 ns ds	600 ns nds
AMYFFD	15.2						15.0									
GPWFWD	16.6															
GVFFFD	15.2															
LIFWYD	15.1															
LIWFFD	14.8							14.9								
LIWWFD_c	15.6													15.4		
LIWWFD_ie	16.2	15.6	16.6	19.1	24.6	16.4								24.3	40.6	16.2
LLFFFD	16.3			16.4												
LLWFFD	15.7	16.5	15.3					15.7	15.8	15.7						
LMWWFD	16.3			16.1												
LPFFFD	15.1															
LVYWFD	15.0															
MIFFFE_c	14.8															
MIFFFE_ie	21.1	31.0	16.1								22.8	37.0	15.6			
MVWFFD	15.3							15.4								
PAFFWD	14.7						15.0									
PIFFWD	14.8															
VLFFFE	14.9															
VVFFWD	16.2			16.2												
VVYFFD	16.5			16.3												
apo 2BEG	14.6															

**Table 5:** Secondary structure occurrences [%] during basic MDS.

2BEG Complex with	Parallel β-Sheets	Anti-Parallel β-Sheets	Σ of β-Sheets	3–10 Helix	α-Helix	π (3–14) Helix	Turn	Bend	Coil
LIWFFD	38	1	39	1	0	0	4	21	35
PAFFWD	25	2	27	1	0	0	6	22	44
LVYWFD	28	0	28	3	1	0	5	21	42
VLFFFE	31	2	33	1	0	0	6	21	39
PIFFWD	30	0	30	1	0	0	5	25	39
AMYFFD	30	3	33	1	1	0	5	23	37
MIFFFE_c	33	1	34	1	0	0	5	25	35
MVWFFD	33	2	35	1	0	0	6	23	35
GVFFFD	32	1	33	1	0	0	4	23	39
LIFWYD	30	1	31	1	0	0	5	0,24	39
LIWWFD_c	28	1	29	2	0	0	9	22	38
LPFFFD	33	2	35	2	1	0	5	21	36
LLWFFD	12	1	13	1	0	0	7	36	43
GPWFWD	8	1	9	0	0	0	4	30	57
VVFFWD	7	2	9	0	0	0	5	38	48
LIWWFD_ie	8	0	8	0	0	0	6	36	50
LMWWFD	7	2	9	1	0	0	7	32	51
LLFFFD	8	1	8	0	0	0	7	33	51
VVYFFD	9	2	11	1	0	0	6	35	47
MIFFFE_ie	7	1	8	1	0	0	7	33	51
apo 2BEG	42	1	43	0	0	0	2	20	35

**Table 6:** Salt bridges’ statistics between the same or preceding/succeeding chains of the Aβ-fibril model molecule during basic MDS.

2BEG Complex with	Global SB Occurrence Inside the Same Chains [%]	Global SB Occurrence between Preceding/Succeeding Chains [%]
PIFFWD	368.2	290.4
LIWFFD	341.9	319.5
MVWFFD	335.5	271.3
LPFFFD	281.0	253.7
GVFFFD	274.5	195.4
PAFFWD	261.1	193.7
VVFFWD	245.2	136.7
LVYWFD	234.2	113.7
MIFFFE_c	214.2	129.1
VLFFFE	210.3	88.3
GPWFWD	204.5	93.3
LIFWYD	187.1	95.8
LLFFFD	159.9	106.0
LMWWFD	154.6	39.5
LIWWFD_c	153.0	72.5
LIWWFD_ie	150.2	44.2
LLWFFD	148.5	143.7
AMYFFD	130.7	87.8
VVYFFD	65.7	128.4
MIFFFE_ie	58.4	76.2
apo 2BEG	313.6	238.5

## Data Availability

Not applicable.

## Supplementary Materials

The following supporting information can be downloaded at: https://www.mdpi.com/article/10.3390/ijms23095247/s1.
