# Supplementary material for: Consecutive Aromatic Residues Are Required for Improved Efficacy of β-Sheet Breakers
Source: Int J Mol Sci. 2022 May 8;23(9):5247. doi: 10.3390/ijms23095247 (PMC9102079; doi:10.3390/ijms23095247)
Supplement: Supplementary file 1 [file ijms-23-05247-s001.zip › Suppl. Table 1.pdf]

**Suppl. Table 1 Hydrogen bonds above 20 % between Aβ-fibril model and ligand in the combined replicas from basic MDS.**

| Ligand | H-bond with ligand as donor | H-bond with ligand as acceptor |
|---|---|---|
| AMYFFD | - | - |
| GPWFWD | - | Pro132@O-Lys64@NH 24,4 % |
| GVFFFD | Phe133@NH-Val75@O 20,5 % | Val132@O-Val102@NH 22,3 % |
| LIFWYD | Phe133@NH-Val50@O 24,2 % | Leu131@O-Val50@NH 21,5 % |
| LIWFFD | Phe135@NH-Ile51@O 20,0 % | - |
| LIWWFD_c | Trp134@NE1HE1-Val46@O 37,5 % | Trp134@O-Gly48@NH 31,8 % |
| LIWWFD_ie | - | Trp134@NE1HE1-Val54@O 28,1 % |
| LLFFFD | - | - |
| LLWFFD | - | - |
| LMWWFD | - | Trp134@O-Gly47@NH 20,5 % |
| LPFFFD | - | - |
| LVYWFD | - | Leu131@O-Phe29@NH 28,9 % |
| MIFFFE_c | - | - |
| MIFFFE_ie | Phe133@NH-Val101@O 23,4 % | - |
| MVWFFD | Trp133@NE1HE1-Phe82@O 29,7 %<br>Phe135@NH-Leu122@O 22,6 % | - |
| PAFFWD | Phe133@NH-Gly48@O 27,0 % | - |
| PIFFWD | Phe134@NH-Val2@O 27,4 % | Phe134@O-Phe4@NH 27,1 % |
| VLFFFE | - | Phe134@O-Gly22@NH 27,4 % |
| VVFFWD | - | - |
| VVYFFD | Phe134@NH-Val127@O 22,8 % | Phe134@O-Ile129@NH 24,5 % |
