# Supplementary figures and images for: Consecutive Aromatic Residues Are Required for Improved Efficacy of β-Sheet Breakers
Source: Int J Mol Sci. 2022 May 8;23(9):5247. doi: 10.3390/ijms23095247 (PMC9102079; doi:10.3390/ijms23095247)

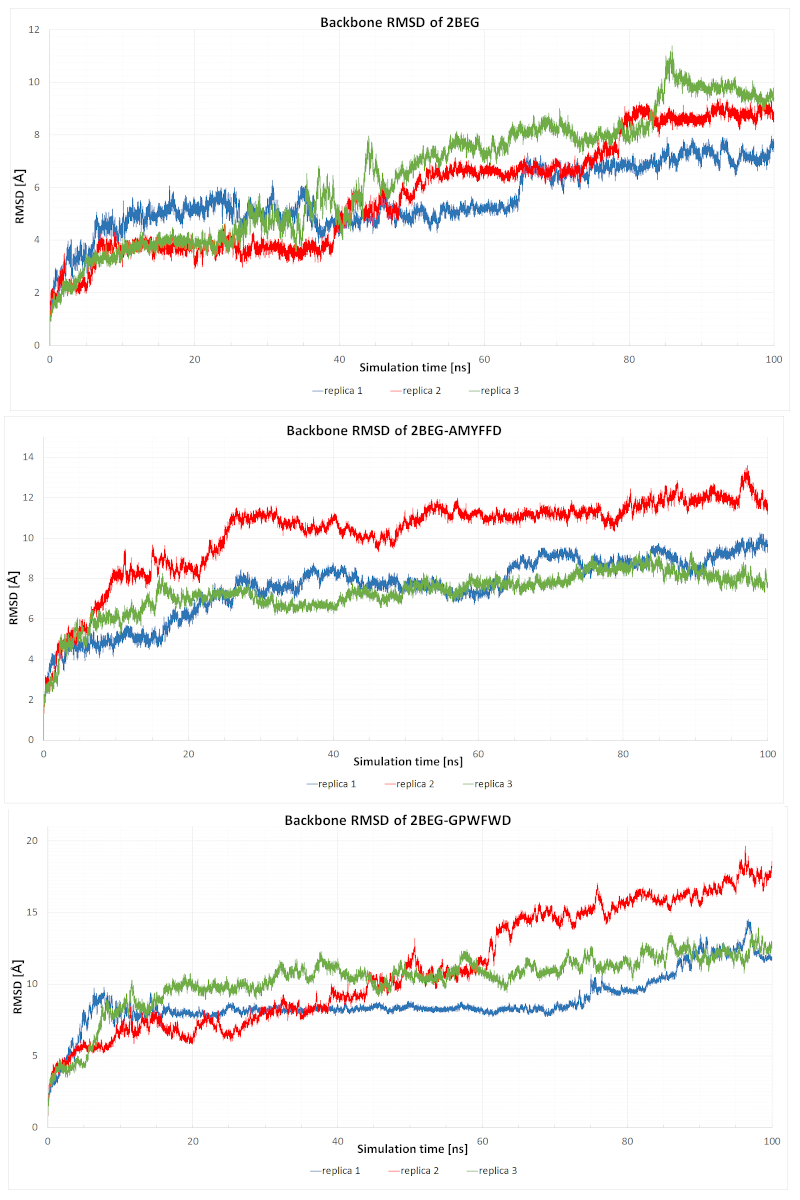

Supplement: Supplementary file 1 [file ijms-23-05247-s001.zip › Suppl. Fig. 1ABC.png]

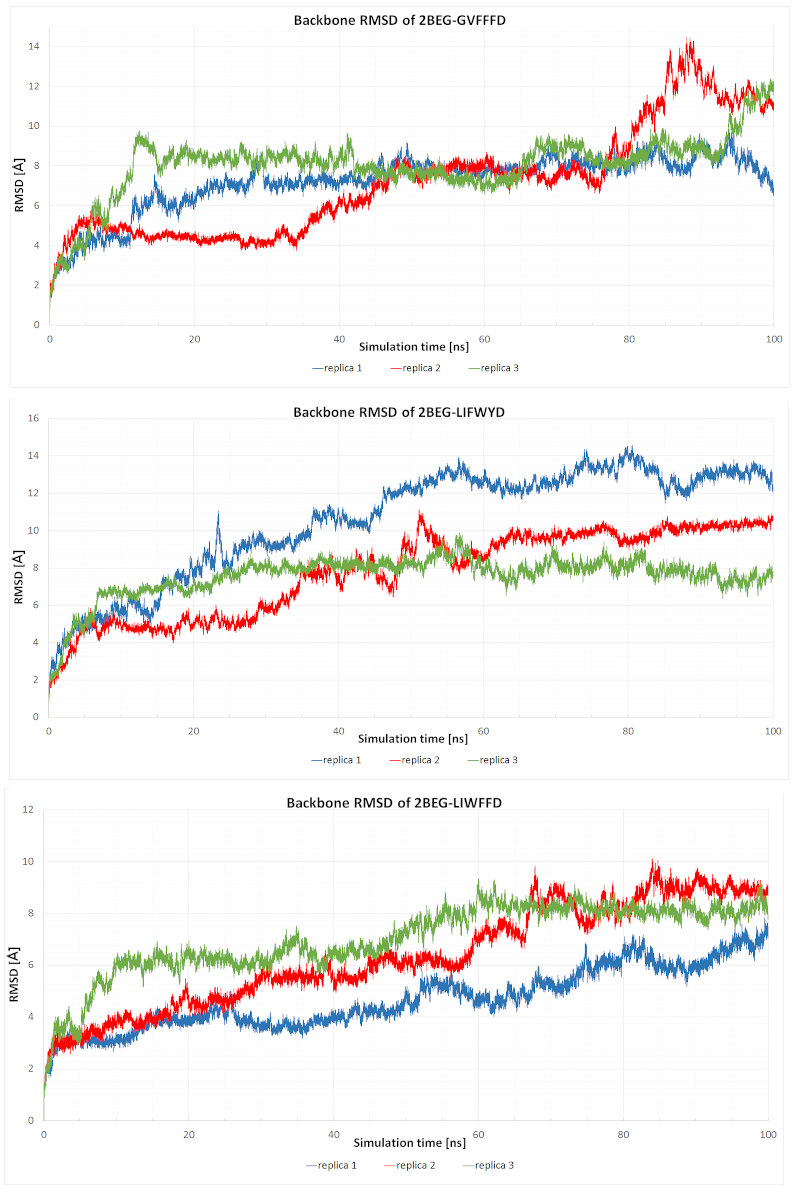

Supplement: Supplementary file 1 [file ijms-23-05247-s001.zip › Suppl. Fig. 1DEF.png]

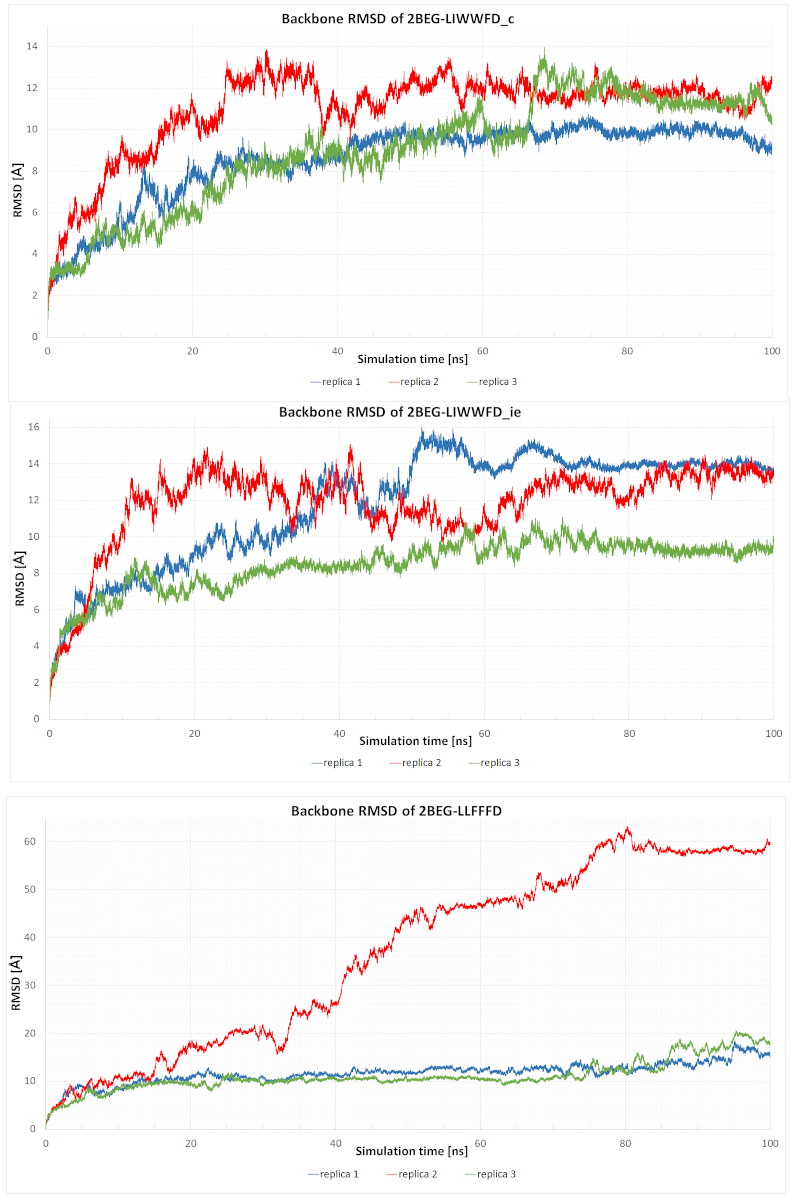

Supplement: Supplementary file 1 [file ijms-23-05247-s001.zip › Suppl. Fig. 1GHI.png]

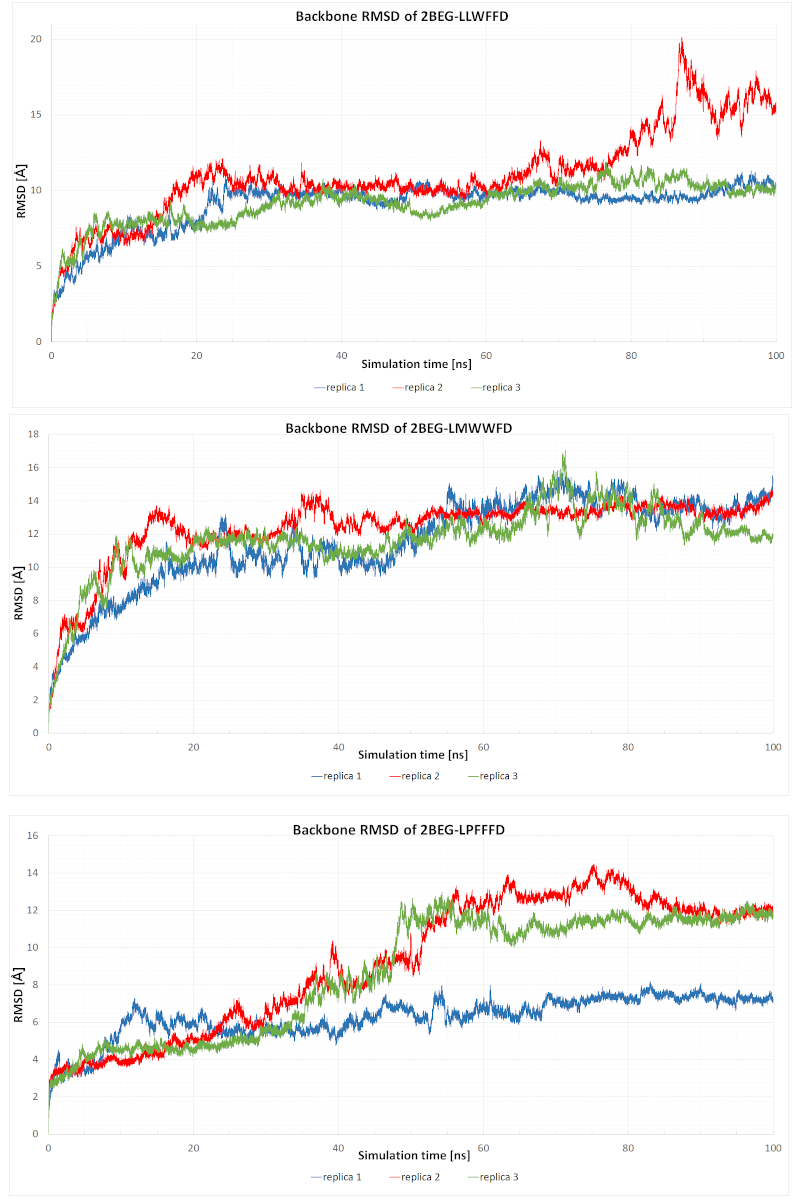

Supplement: Supplementary file 1 [file ijms-23-05247-s001.zip › Suppl. Fig. 1JKL.png]

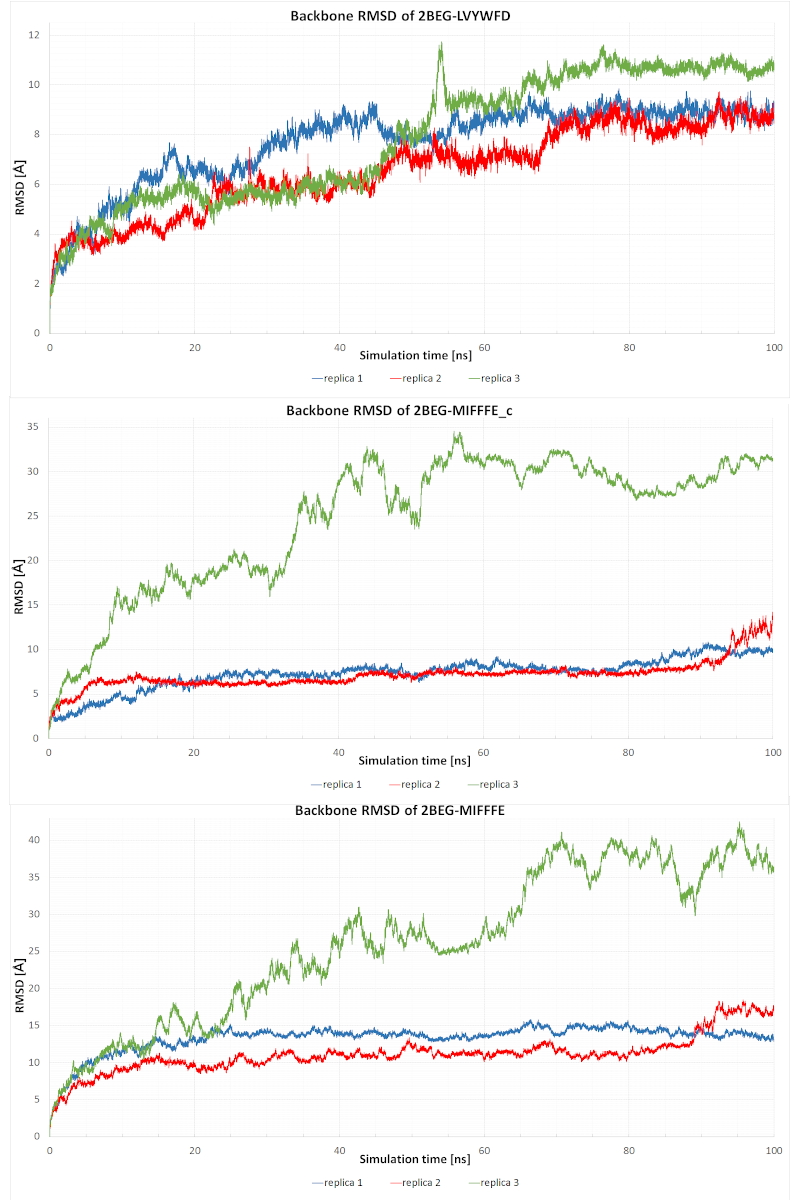

Supplement: Supplementary file 1 [file ijms-23-05247-s001.zip › Suppl. Fig. 1MNO.png]

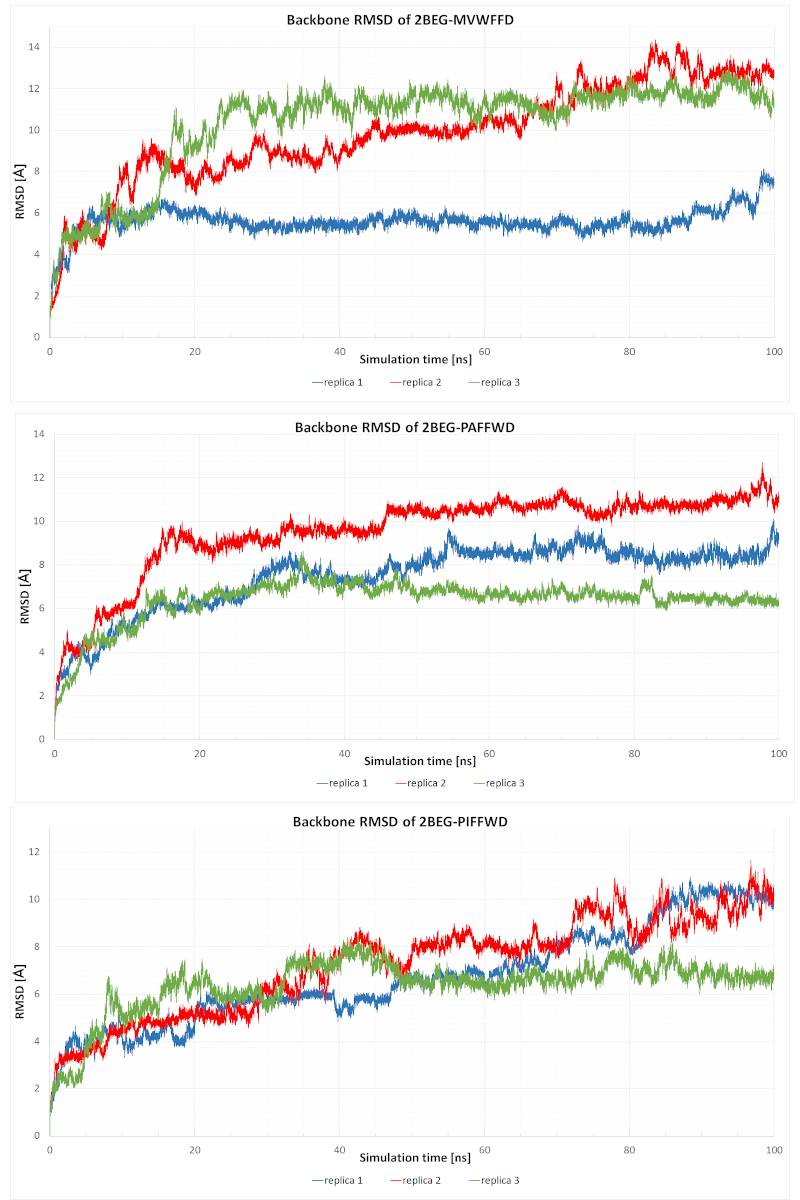

Supplement: Supplementary file 1 [file ijms-23-05247-s001.zip › Suppl. Fig. 1PQR.png]

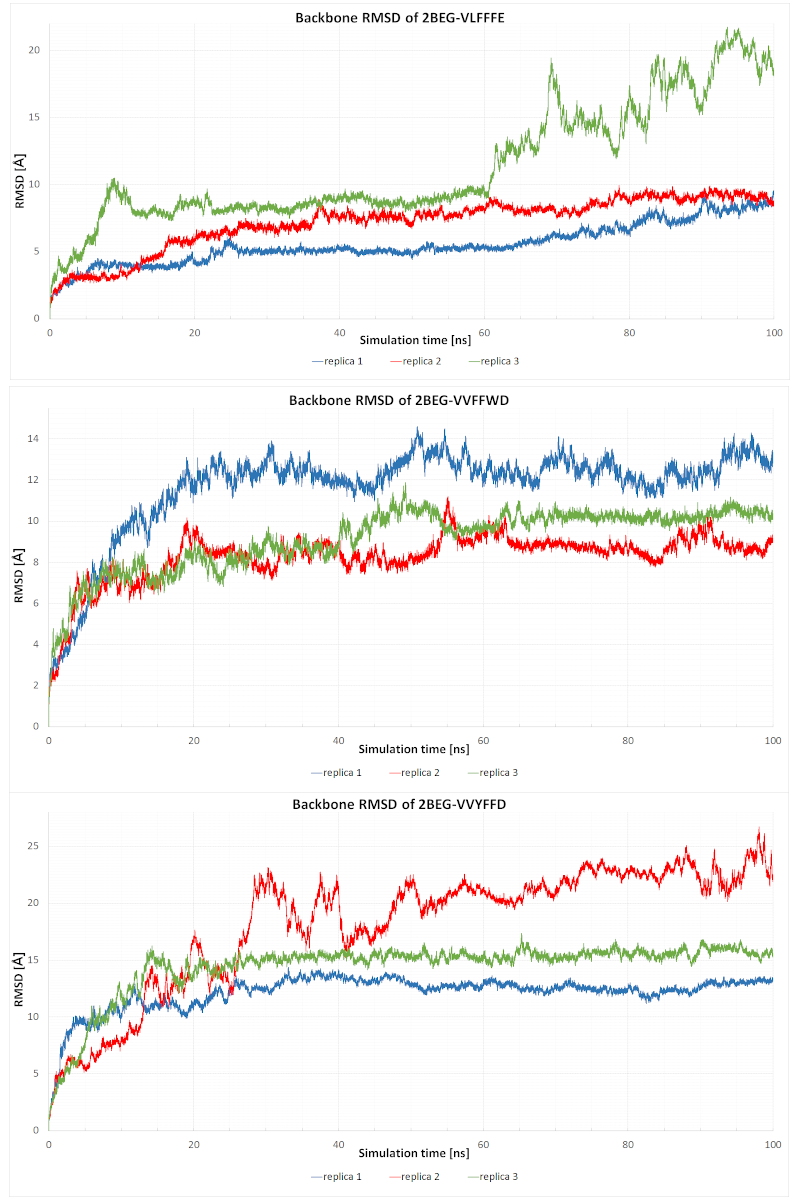

Supplement: Supplementary file 1 [file ijms-23-05247-s001.zip › Suppl. Fig. 1STU.png]

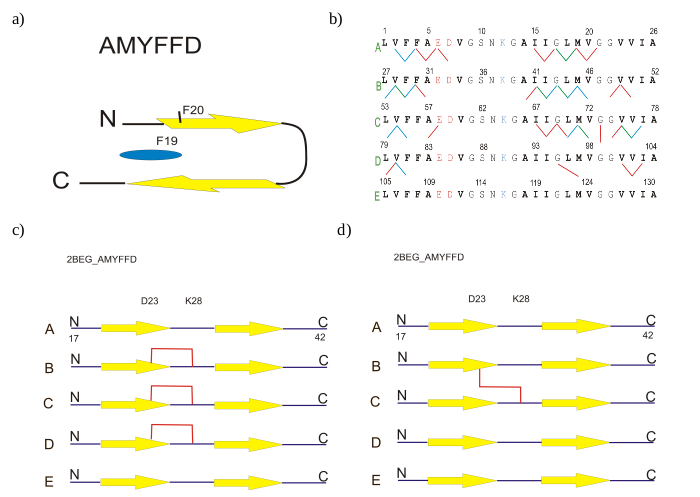

Supplement: Supplementary file 1 [file ijms-23-05247-s001.zip › Suppl. Fig. 2A AMYFFD.png]

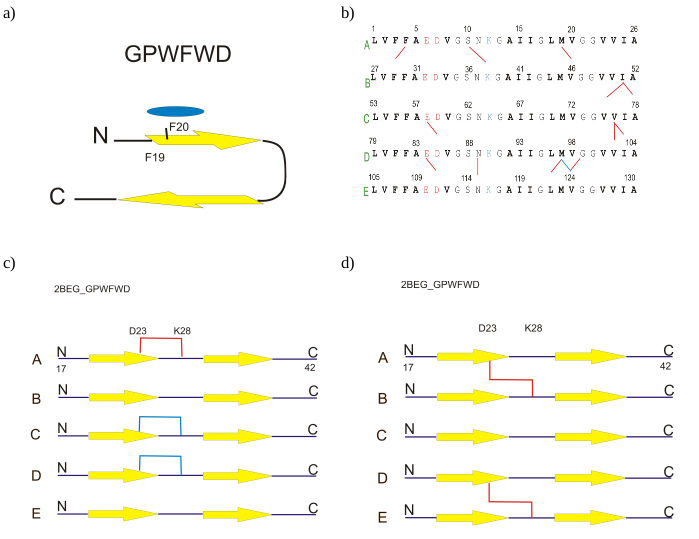

Supplement: Supplementary file 1 [file ijms-23-05247-s001.zip › Suppl. Fig. 2B GPWFWD.png]

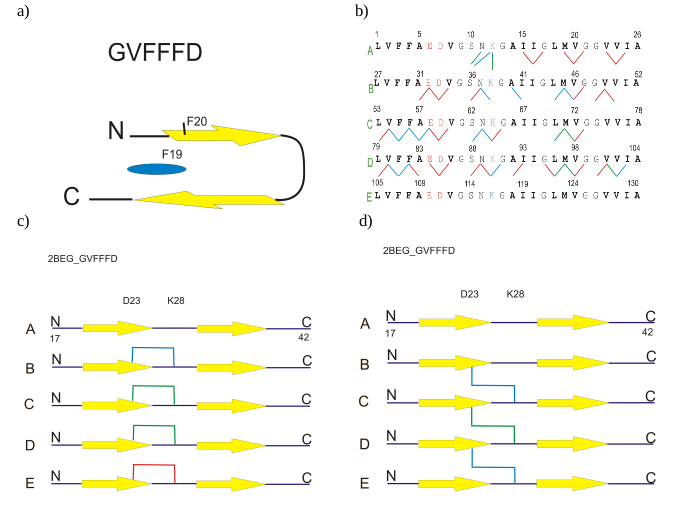

Supplement: Supplementary file 1 [file ijms-23-05247-s001.zip › Suppl. Fig. 2C GVFFFD.png]

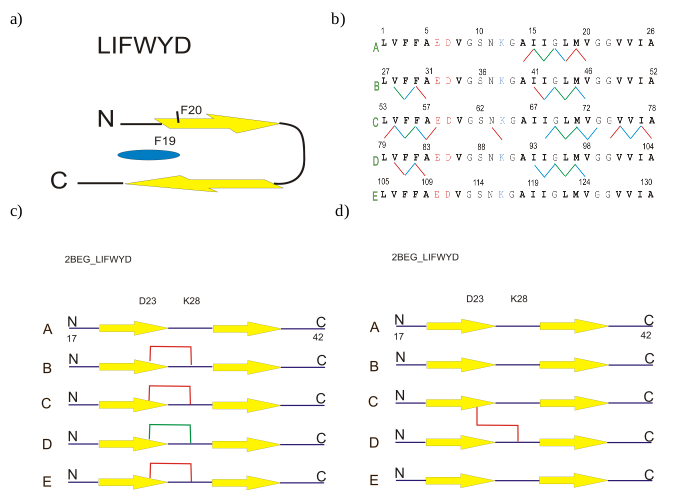

Supplement: Supplementary file 1 [file ijms-23-05247-s001.zip › Suppl. Fig. 2D LIFWYD.png]

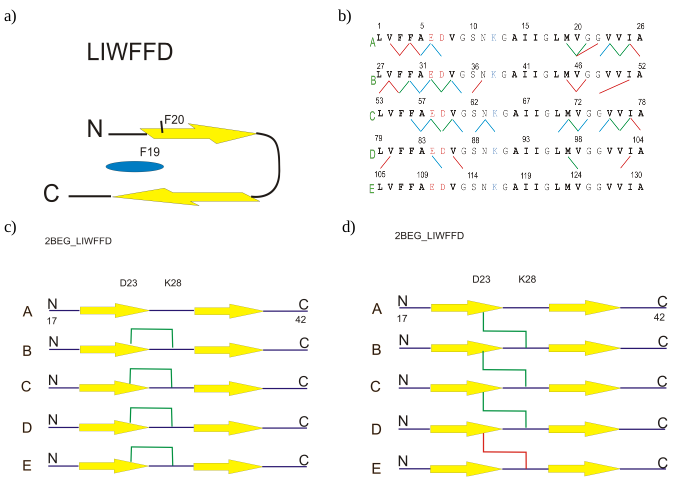

Supplement: Supplementary file 1 [file ijms-23-05247-s001.zip › Suppl. Fig. 2E LIWFFD.png]

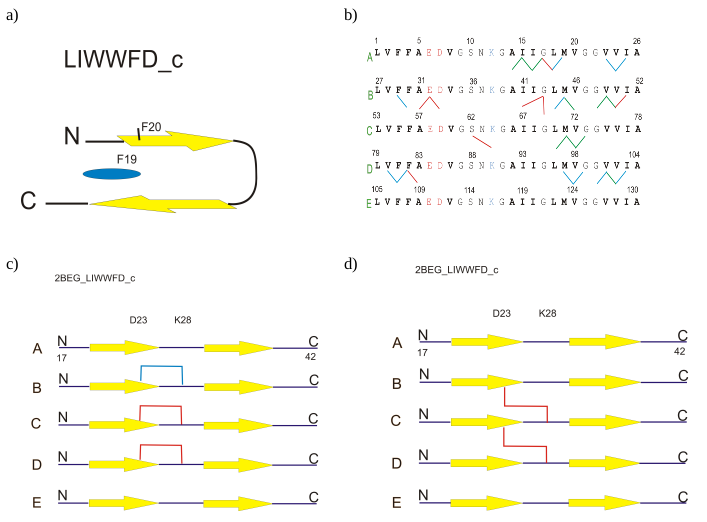

Supplement: Supplementary file 1 [file ijms-23-05247-s001.zip › Suppl. Fig. 2F LIWWFD_c.png]

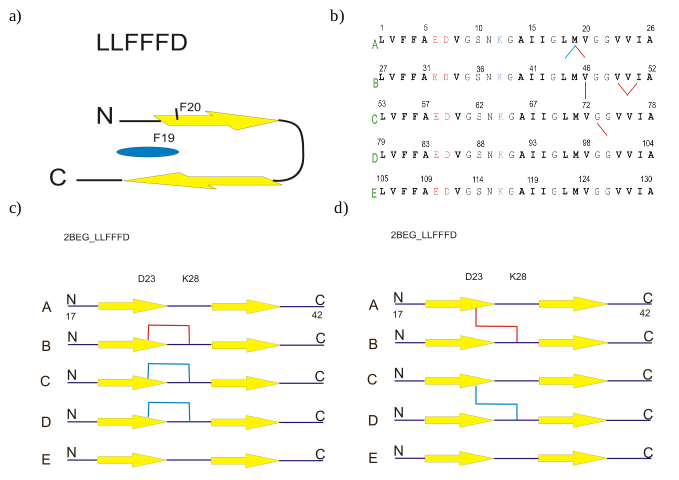

Supplement: Supplementary file 1 [file ijms-23-05247-s001.zip › Suppl. Fig. 2G LLFFFD.png]

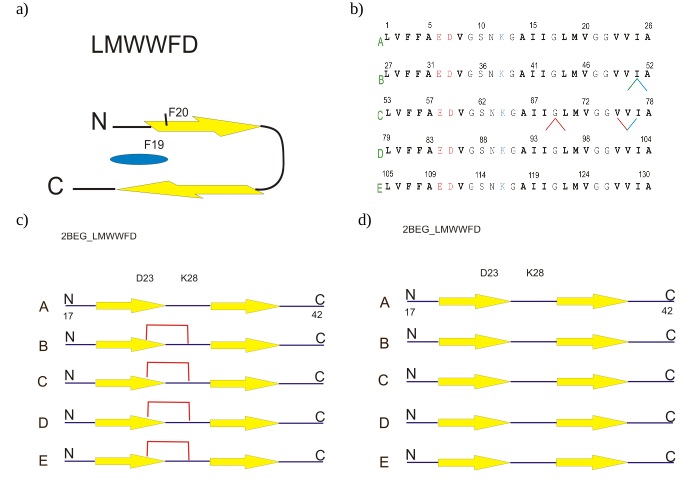

Supplement: Supplementary file 1 [file ijms-23-05247-s001.zip › Suppl. Fig. 2H LMWWFD.png]

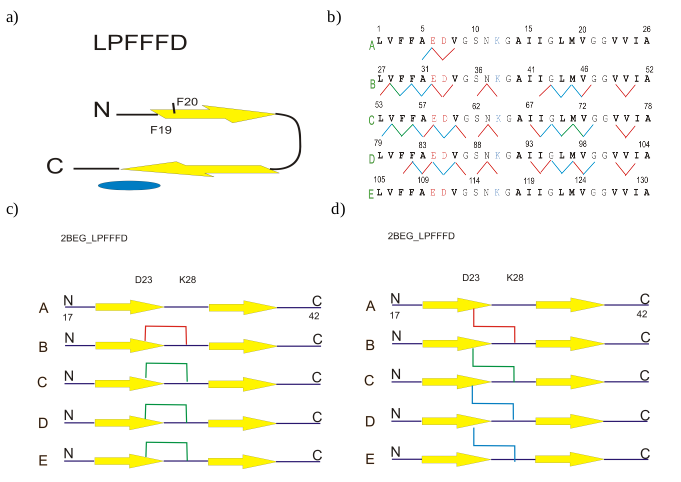

Supplement: Supplementary file 1 [file ijms-23-05247-s001.zip › Suppl. Fig. 2I LPFFFD.png]

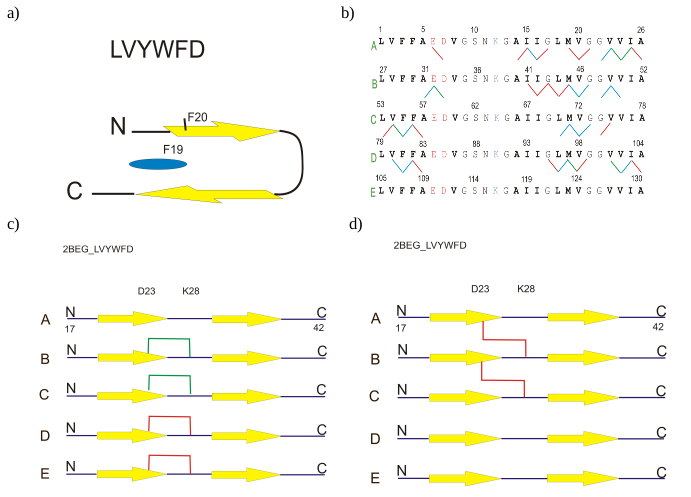

Supplement: Supplementary file 1 [file ijms-23-05247-s001.zip › Suppl. Fig. 2J LVYWFD.png]

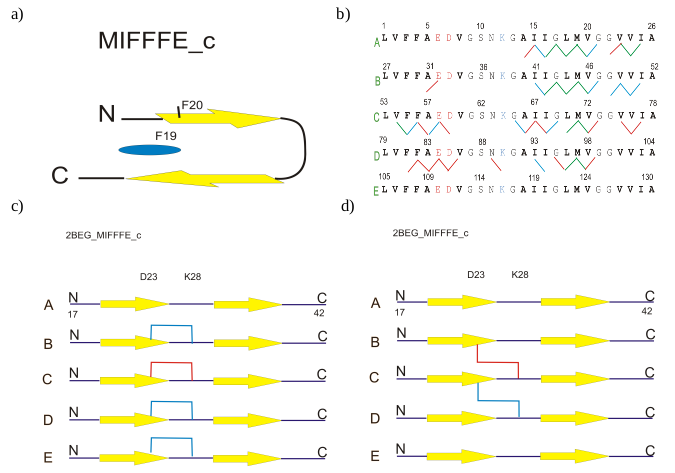

Supplement: Supplementary file 1 [file ijms-23-05247-s001.zip › Suppl. Fig. 2K MIFFFE_c.png]

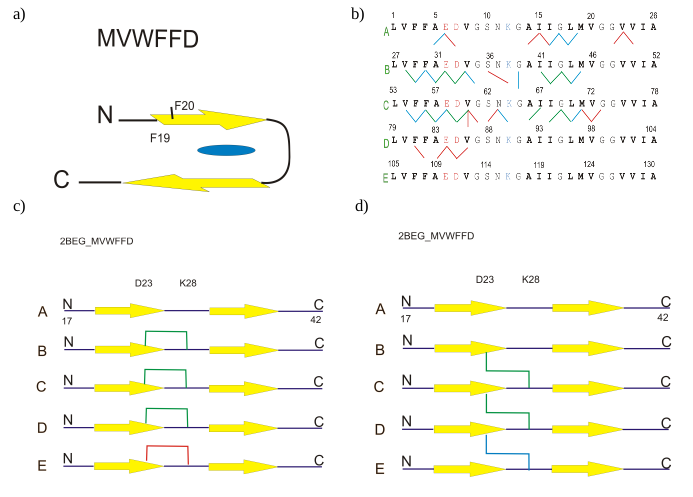

Supplement: Supplementary file 1 [file ijms-23-05247-s001.zip › Suppl. Fig. 2L MVWFFD.png]

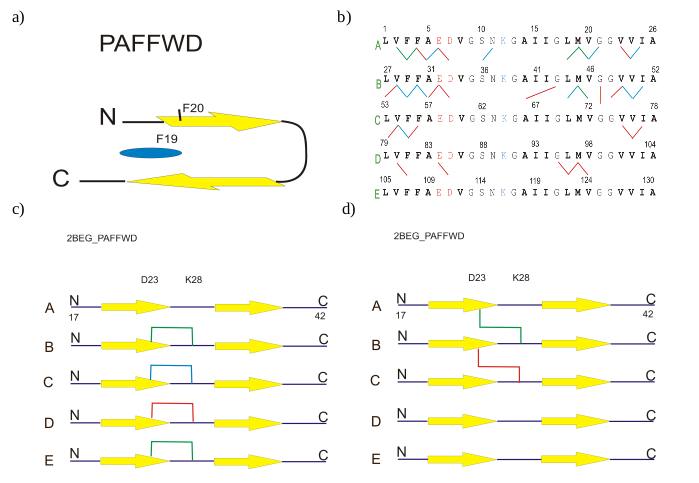

Supplement: Supplementary file 1 [file ijms-23-05247-s001.zip › Suppl. Fig. 2M PAFFWD.png]

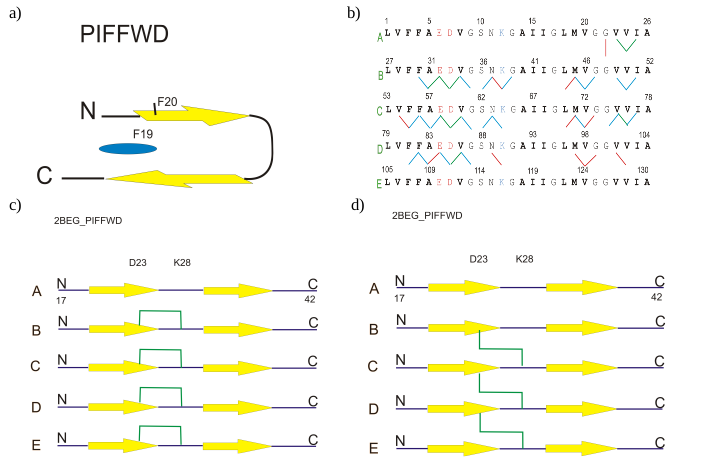

Supplement: Supplementary file 1 [file ijms-23-05247-s001.zip › Suppl. Fig. 2N PIFFWD.png]

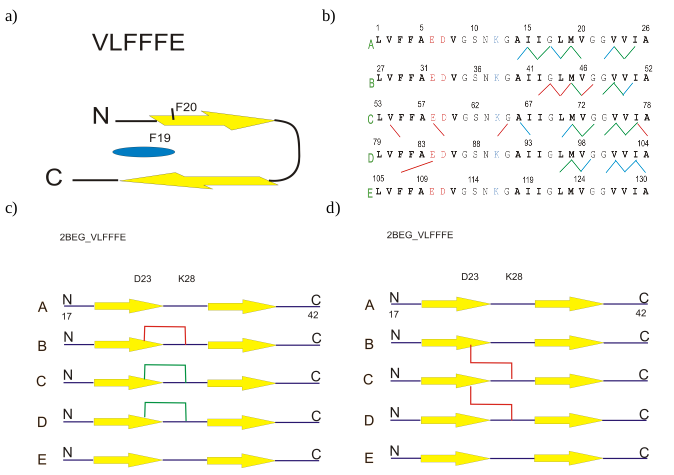

Supplement: Supplementary file 1 [file ijms-23-05247-s001.zip › Suppl. Fig. 2O VLFFFE.png]

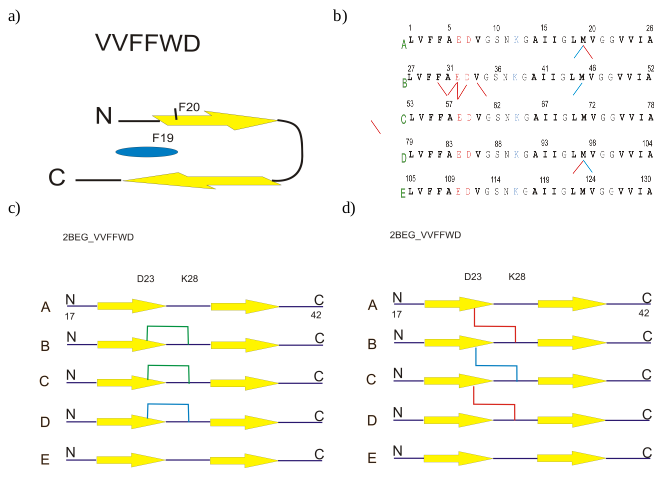

Supplement: Supplementary file 1 [file ijms-23-05247-s001.zip › Suppl. Fig. 2P VVFFWD.png]

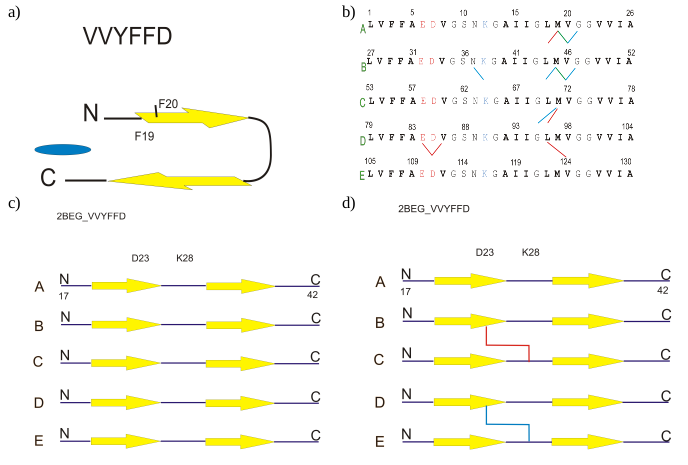

Supplement: Supplementary file 1 [file ijms-23-05247-s001.zip › Suppl. Fig. 2Q VVYFFD.png]

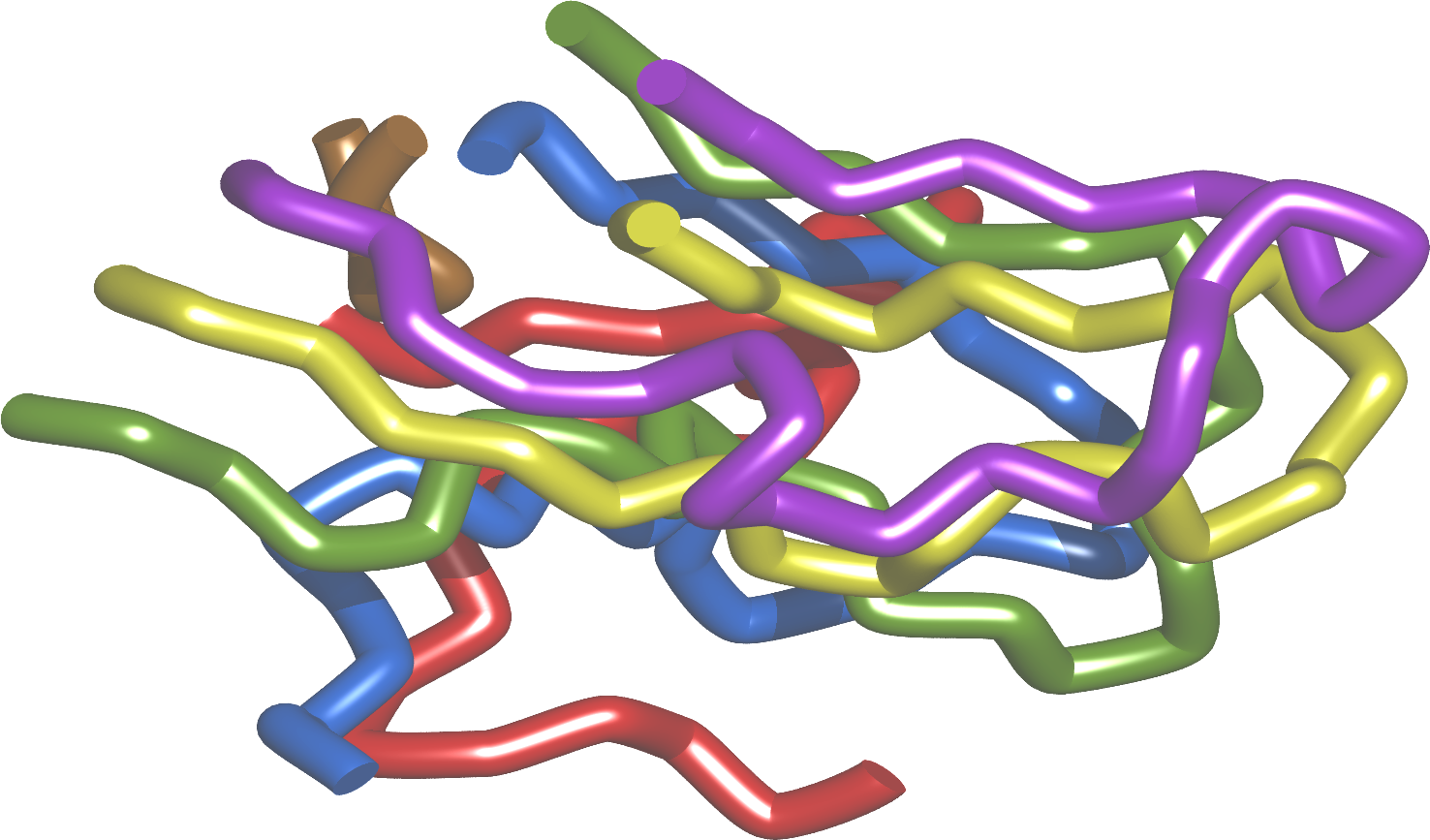

Supplement: Supplementary file 1 [file ijms-23-05247-s001.zip › Suppl. Fig. 3A AMYFFD_13_new.png]

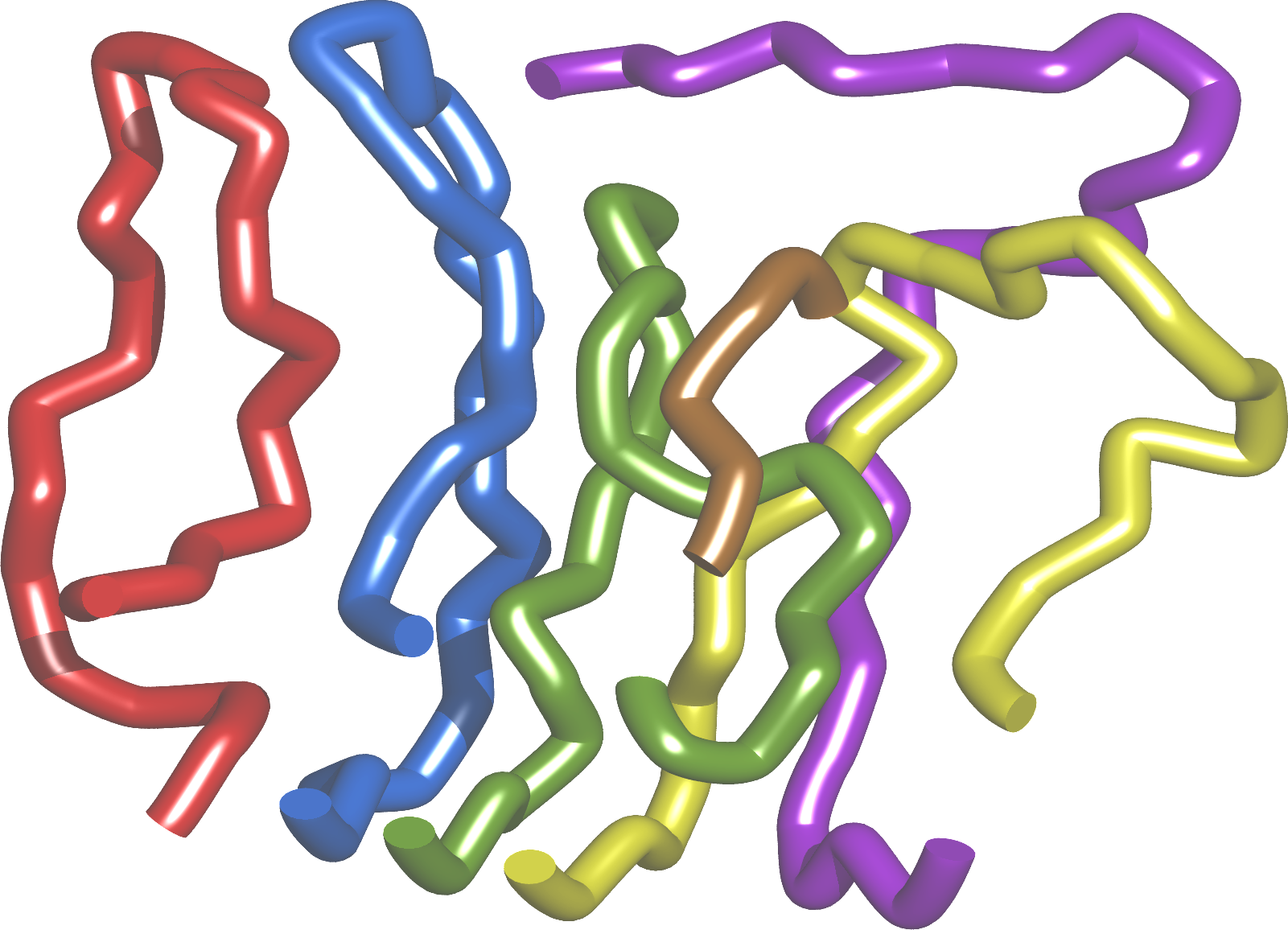

Supplement: Supplementary file 1 [file ijms-23-05247-s001.zip › Suppl. Fig. 3B GPWFWD_cluster_repr_1-3_c0.png]

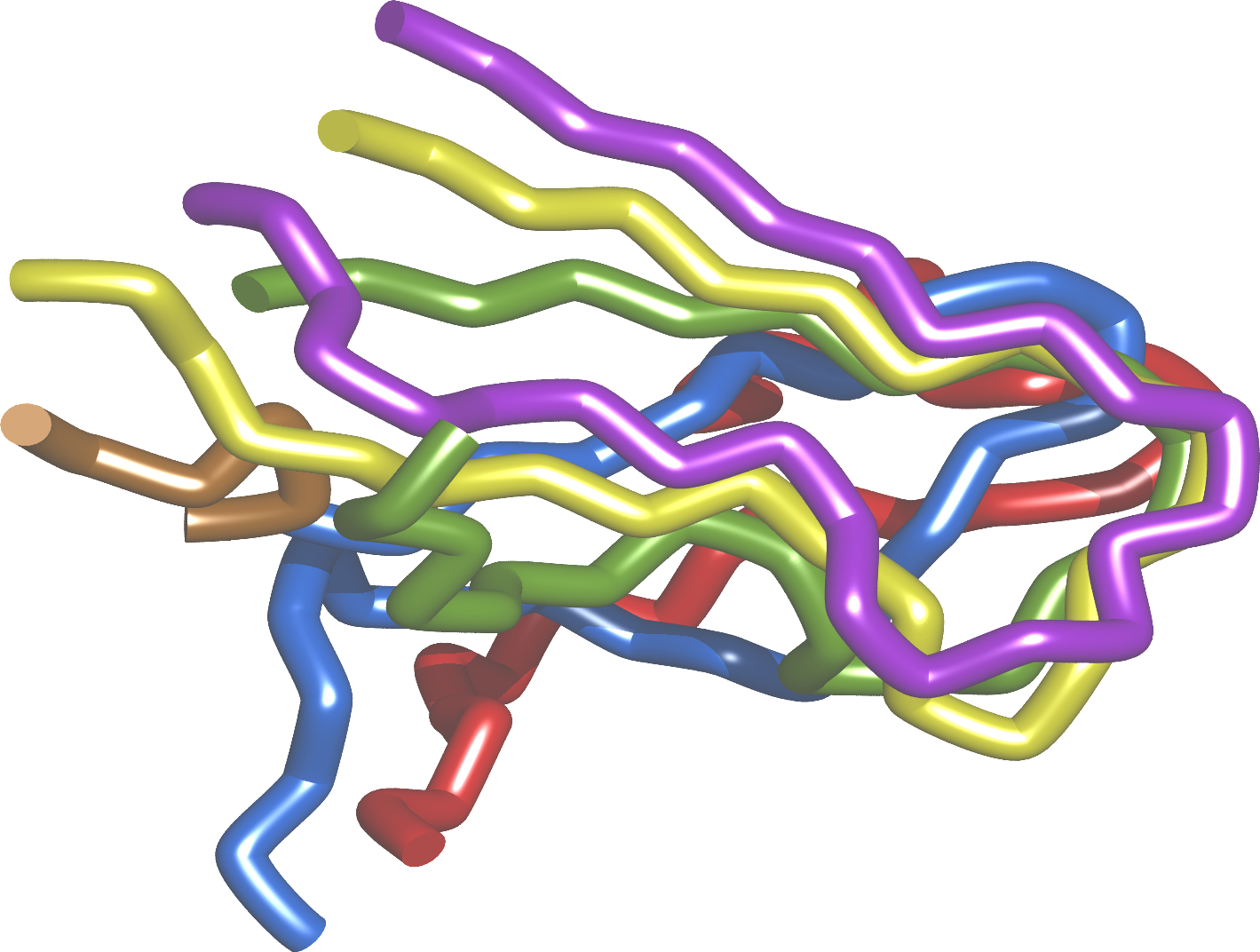

Supplement: Supplementary file 1 [file ijms-23-05247-s001.zip › Suppl. Fig. 3C GVFFFD_13_new.png]

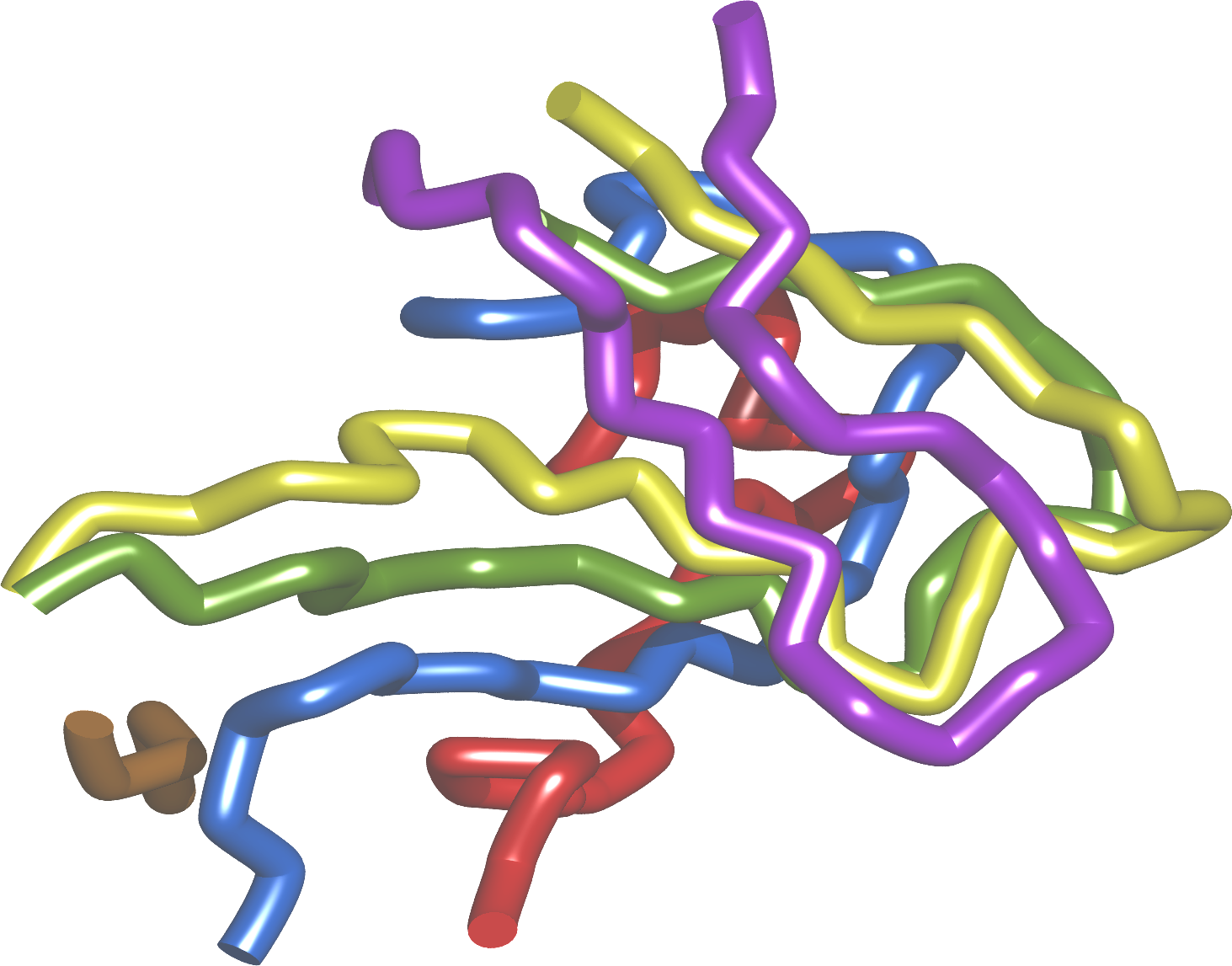

Supplement: Supplementary file 1 [file ijms-23-05247-s001.zip › Suppl. Fig. 3D LIFWYD_13_new.png]

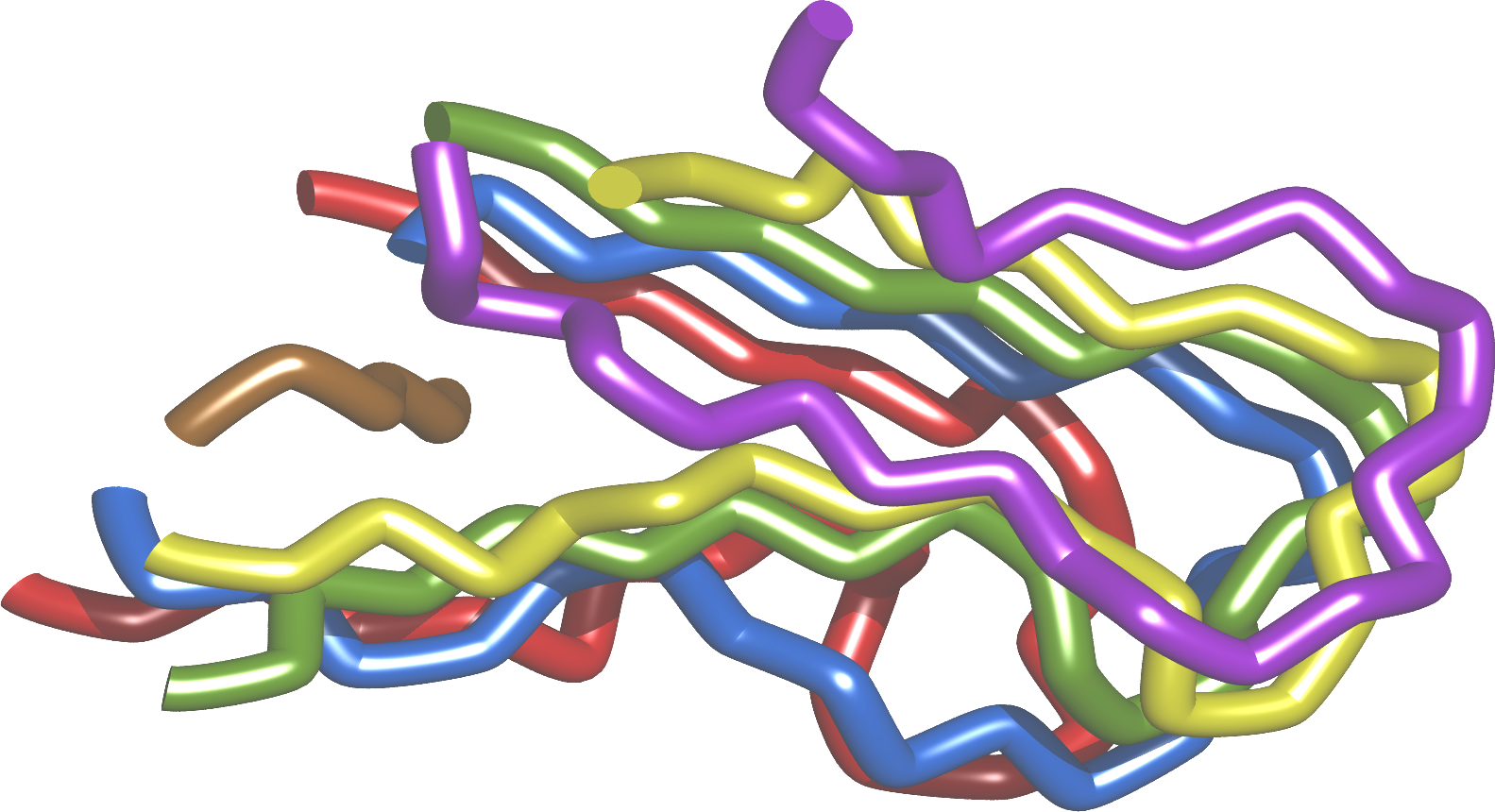

Supplement: Supplementary file 1 [file ijms-23-05247-s001.zip › Suppl. Fig. 3E LIWFFD_13_new.png]

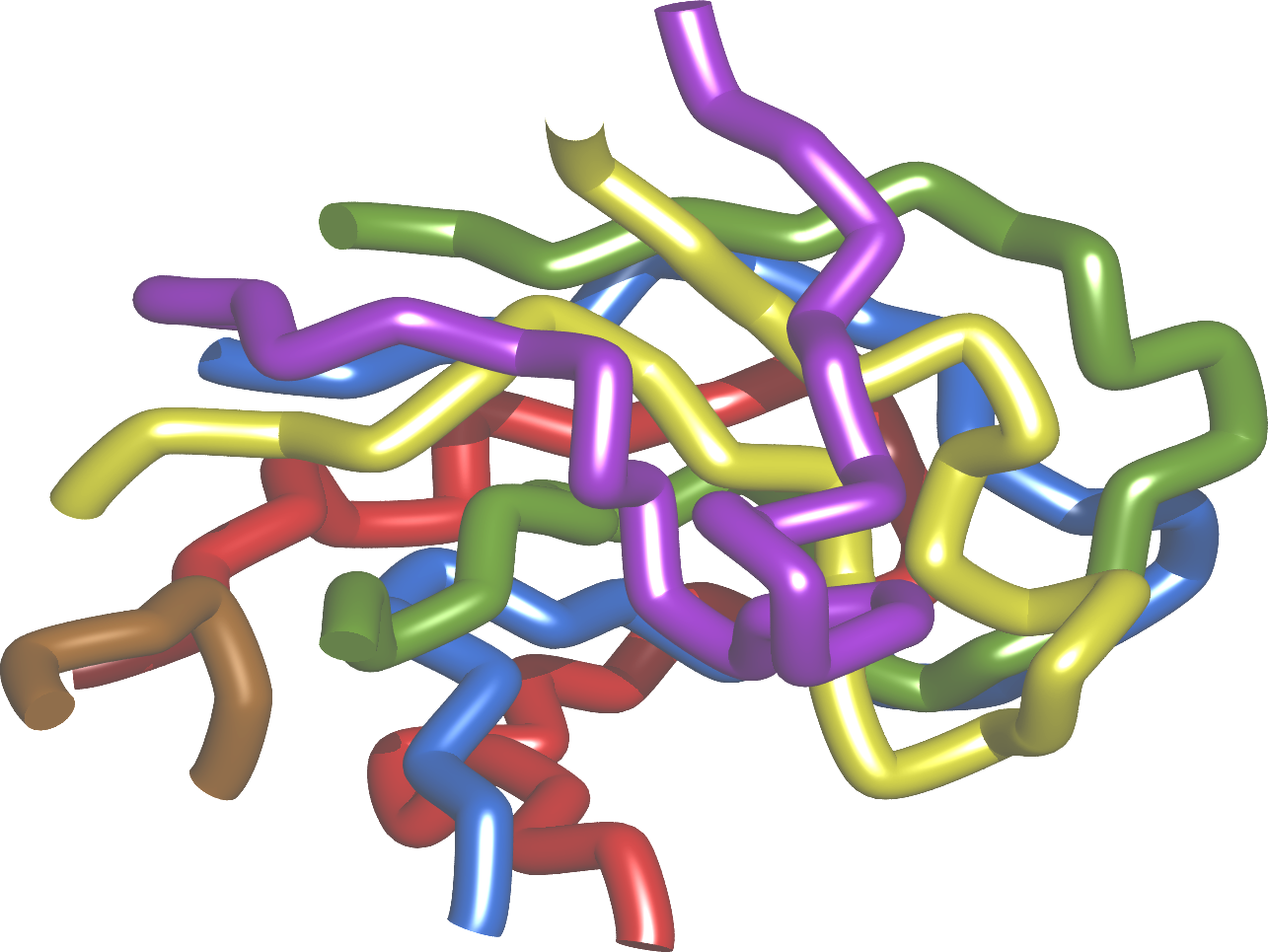

Supplement: Supplementary file 1 [file ijms-23-05247-s001.zip › Suppl. Fig. 3F LIWWFD_c_13_new.png]

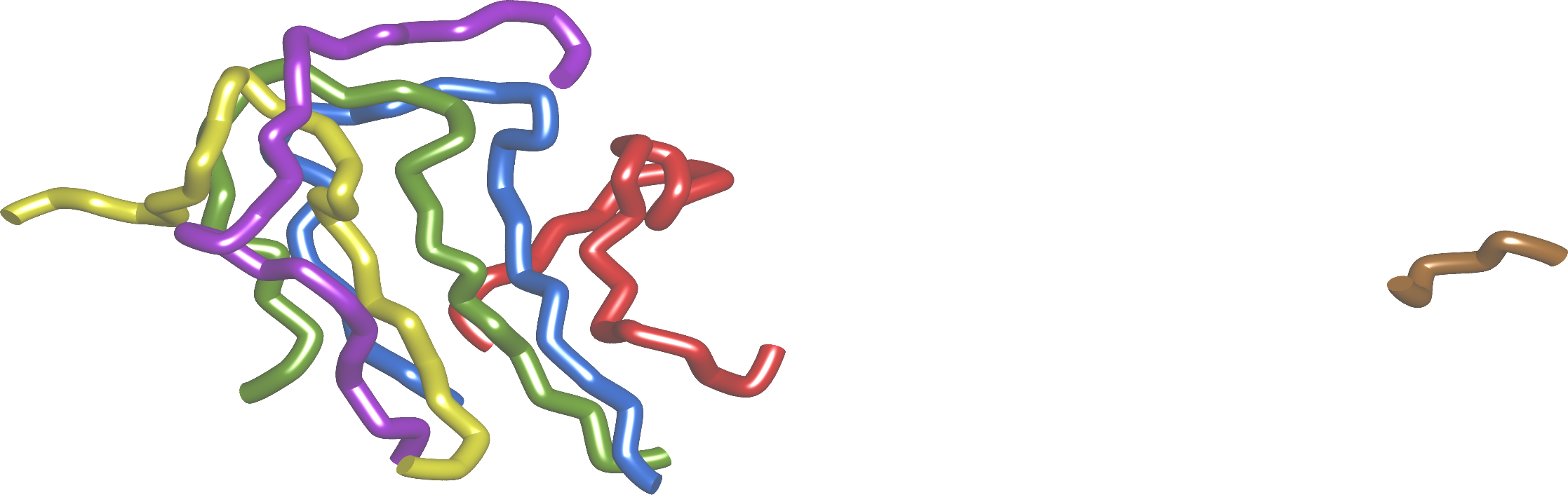

Supplement: Supplementary file 1 [file ijms-23-05247-s001.zip › Suppl. Fig. 3G LLFFFD_cluster_repr_1-3_c2.png]

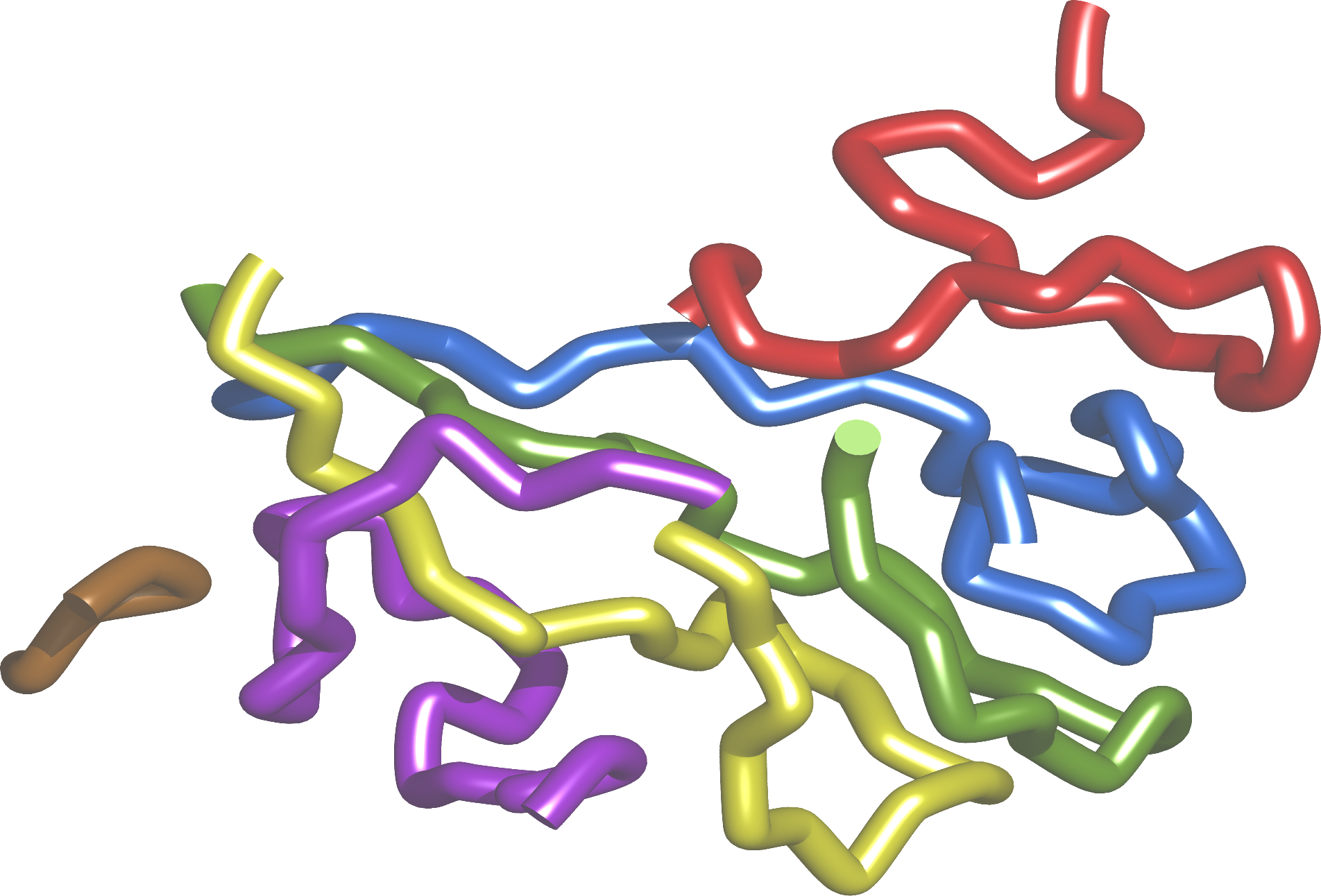

Supplement: Supplementary file 1 [file ijms-23-05247-s001.zip › Suppl. Fig. 3H LMWWFD_cluster_repr_1-3_c0.png]

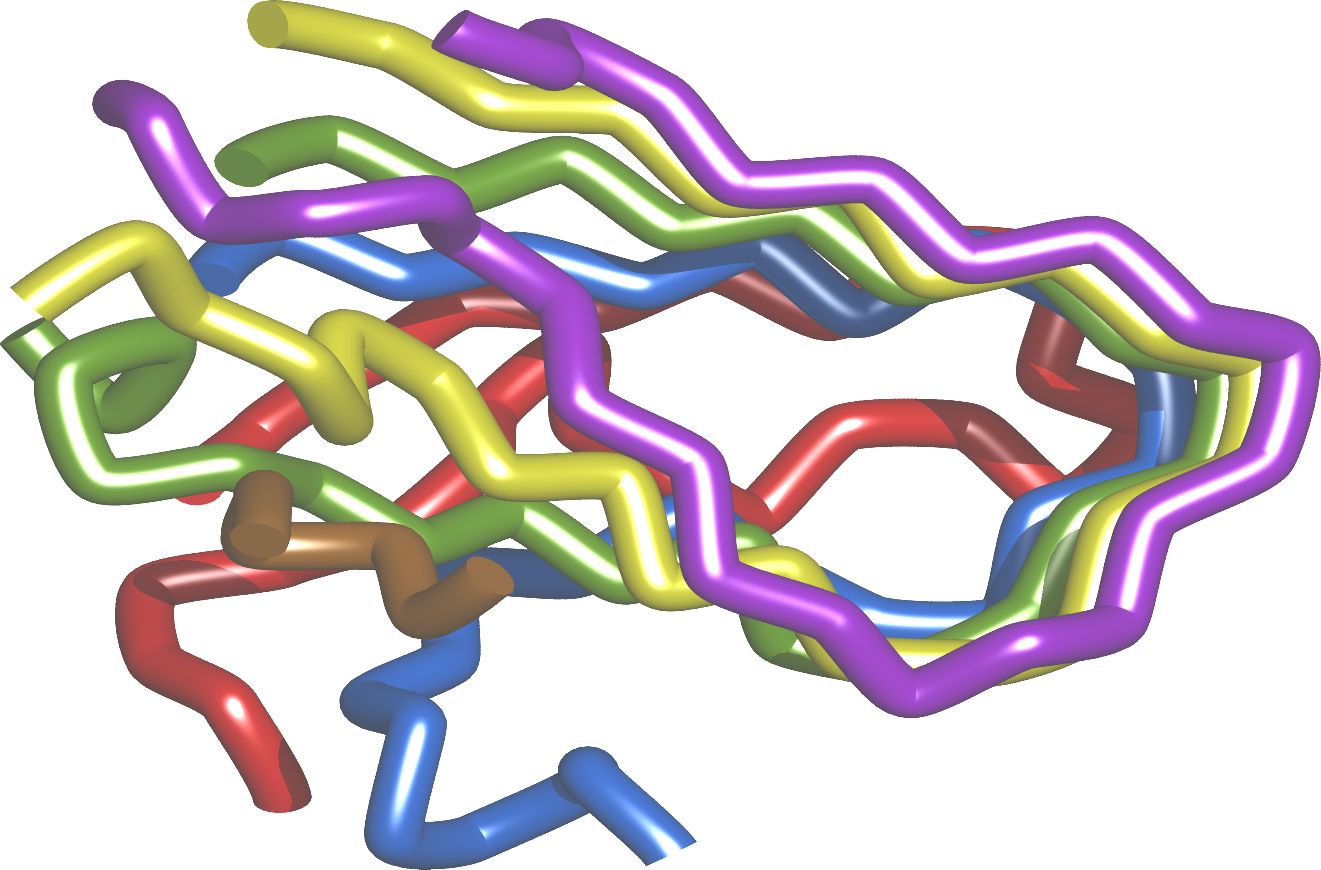

Supplement: Supplementary file 1 [file ijms-23-05247-s001.zip › Suppl. Fig. 3I LPFFFD_13_new.png]

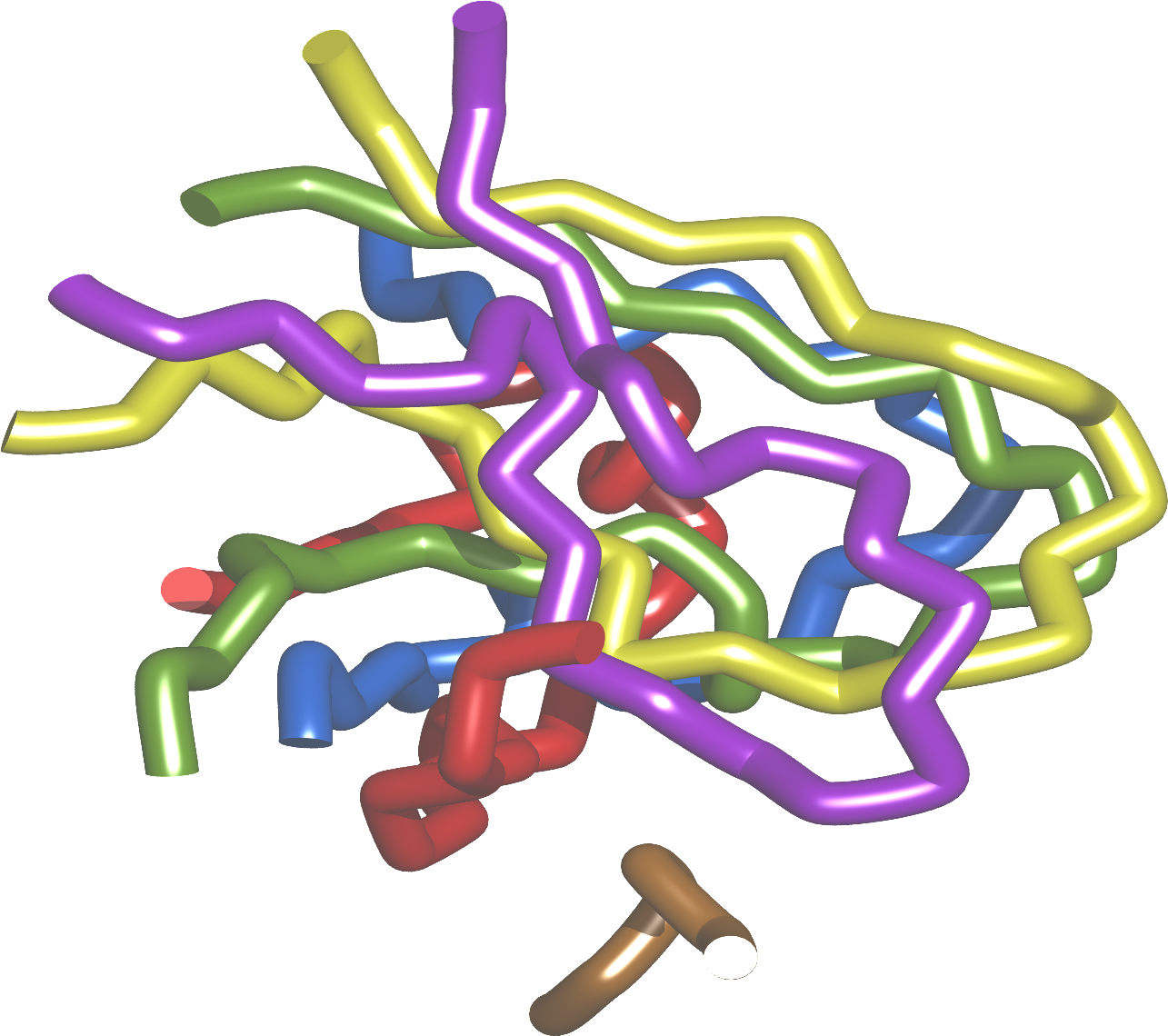

Supplement: Supplementary file 1 [file ijms-23-05247-s001.zip › Suppl. Fig. 3J LVYWFD_13_new.png]

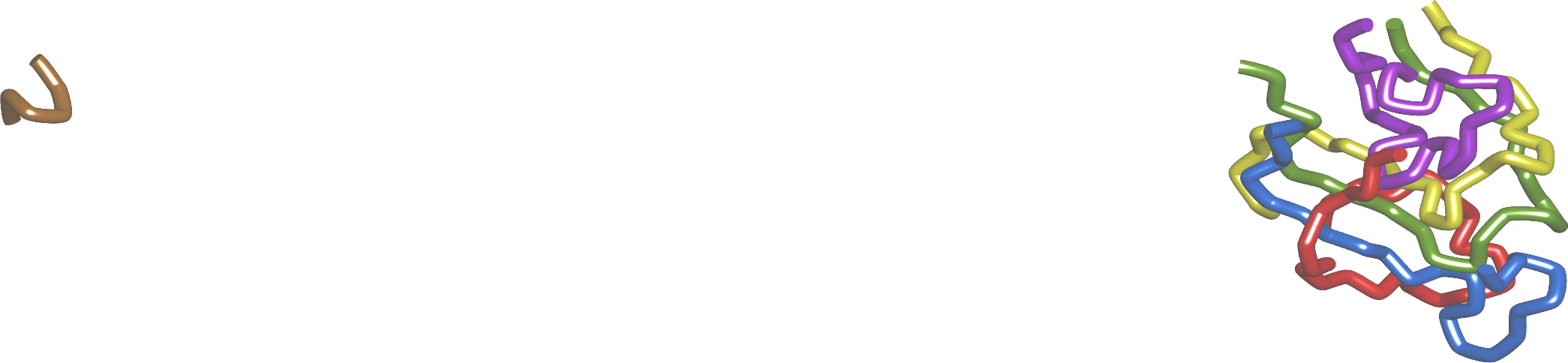

Supplement: Supplementary file 1 [file ijms-23-05247-s001.zip › Suppl. Fig. 3K MIFFFE_c_cluster_repr_1-3_c2.png]

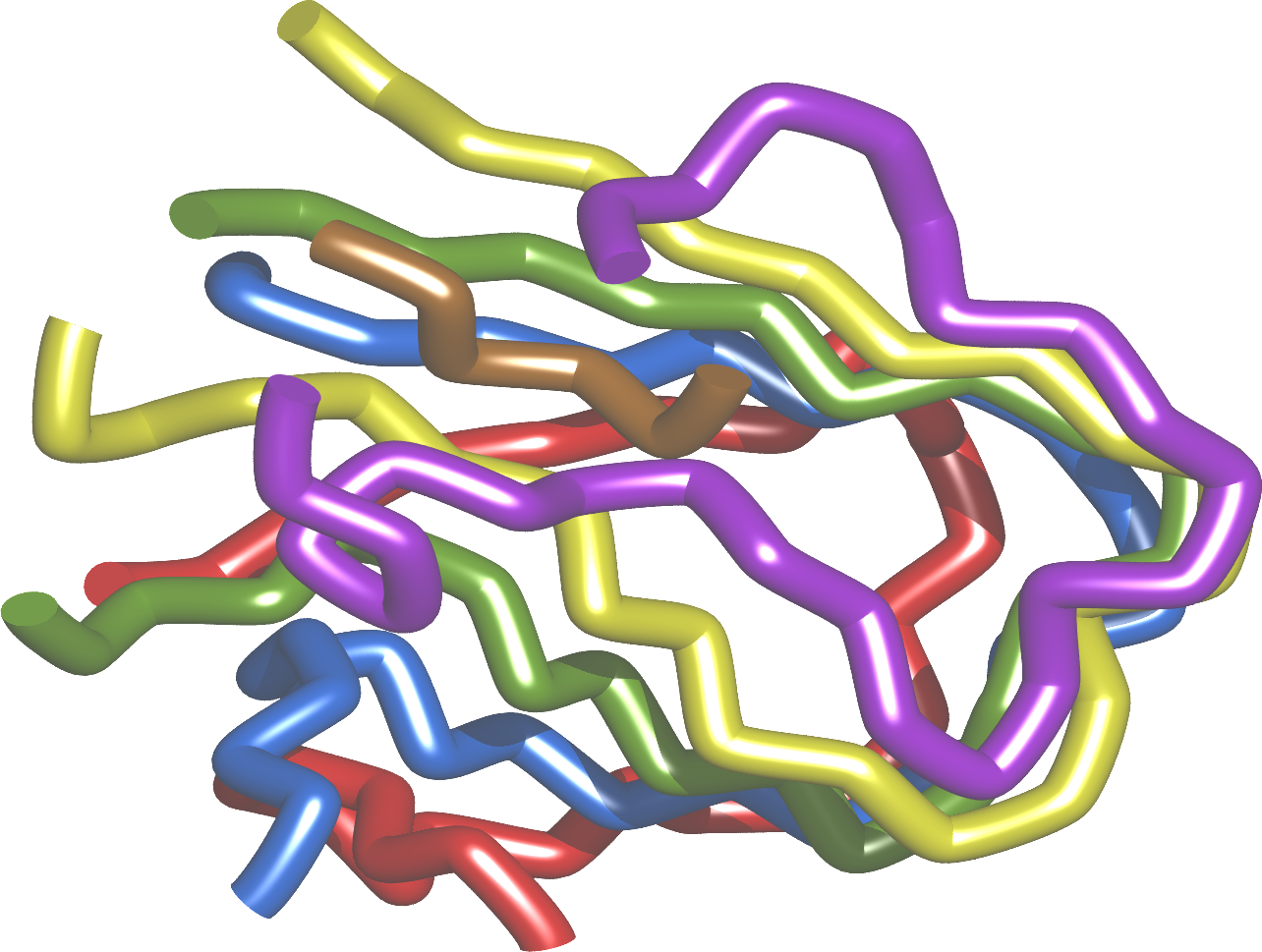

Supplement: Supplementary file 1 [file ijms-23-05247-s001.zip › Suppl. Fig. 3L MVWFFD_13_new.png]

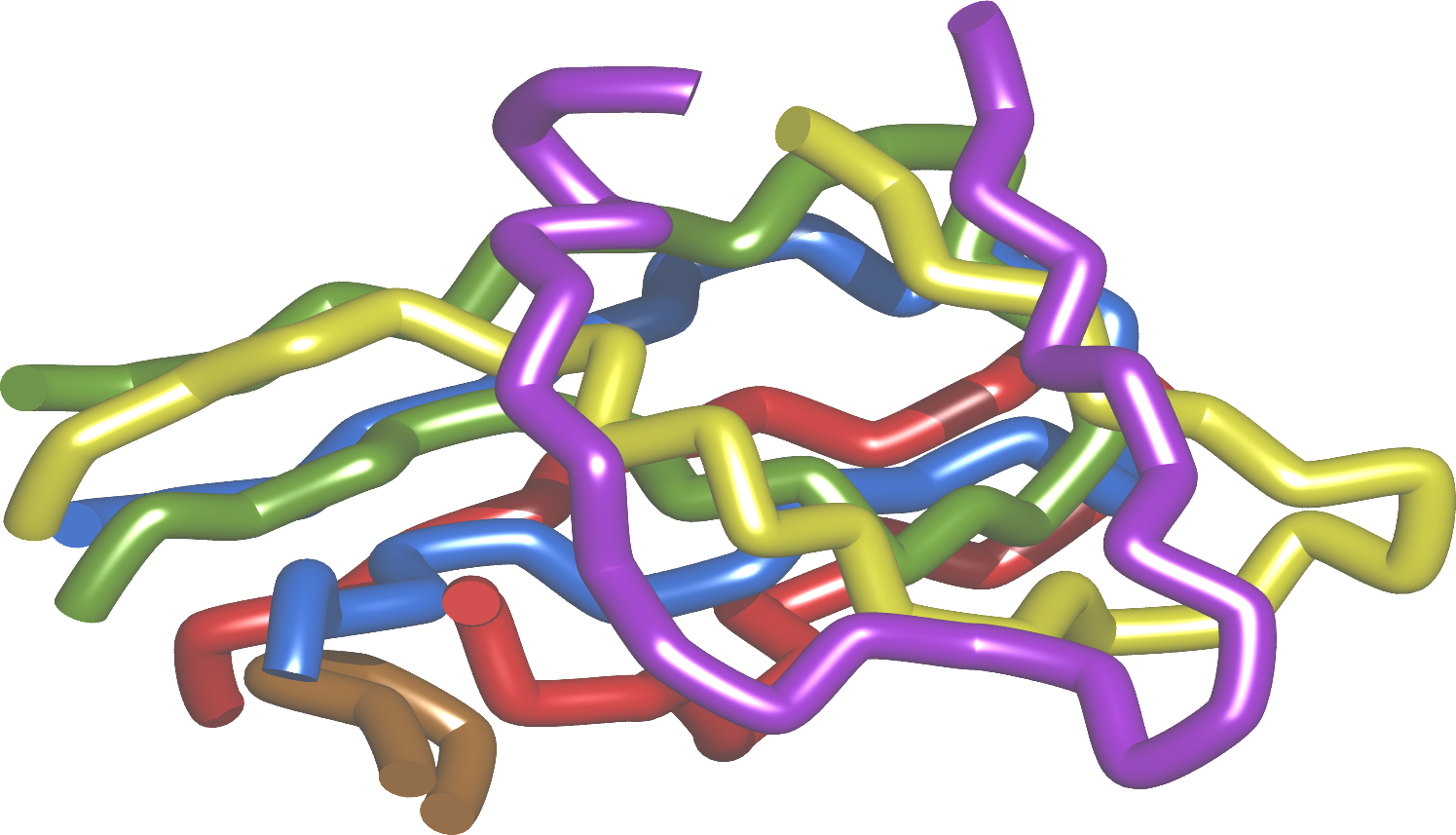

Supplement: Supplementary file 1 [file ijms-23-05247-s001.zip › Suppl. Fig. 3M PAFFWD_13_new.png]

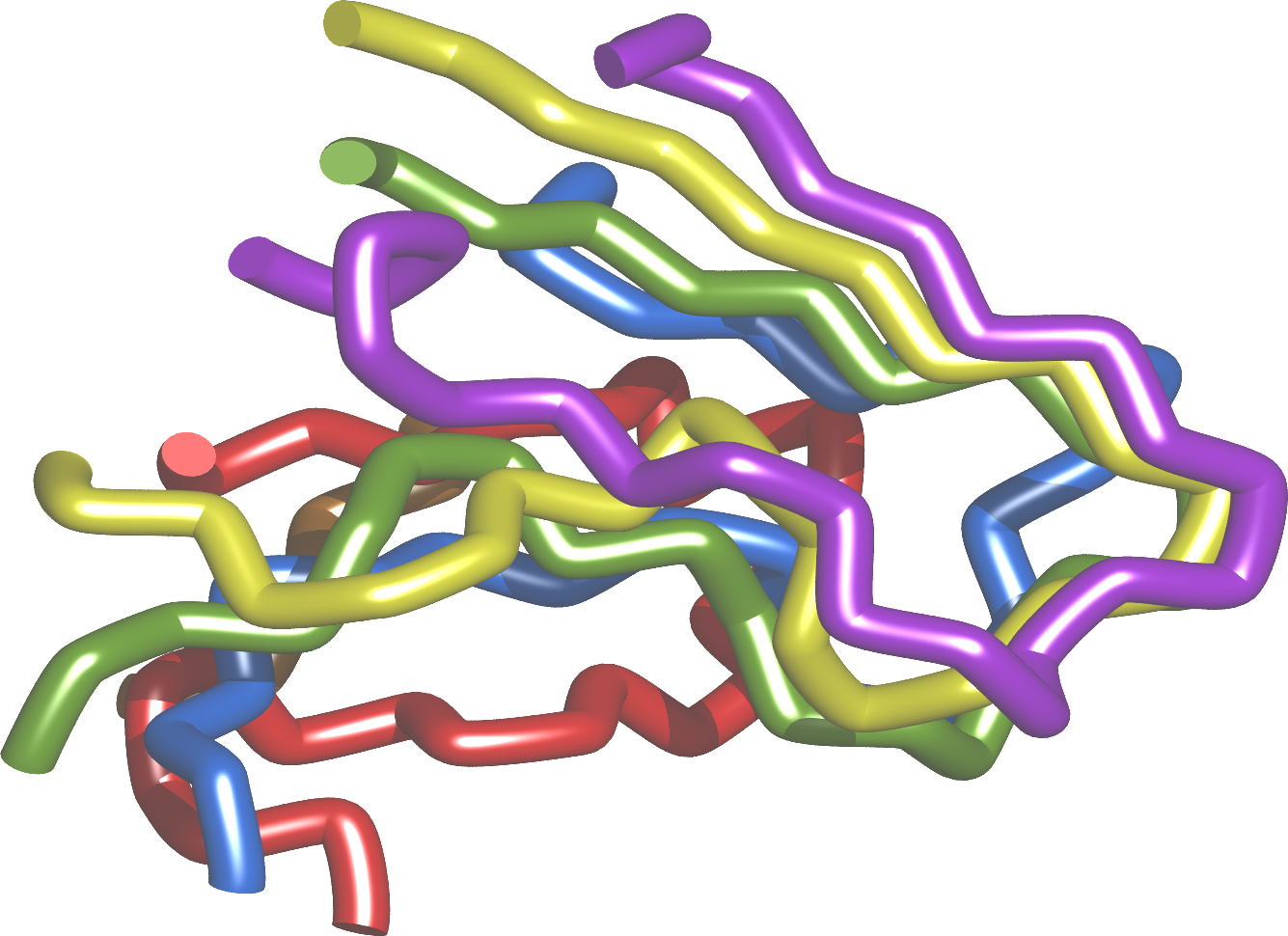

Supplement: Supplementary file 1 [file ijms-23-05247-s001.zip › Suppl. Fig. 3N PIFFWD_13_new.png]

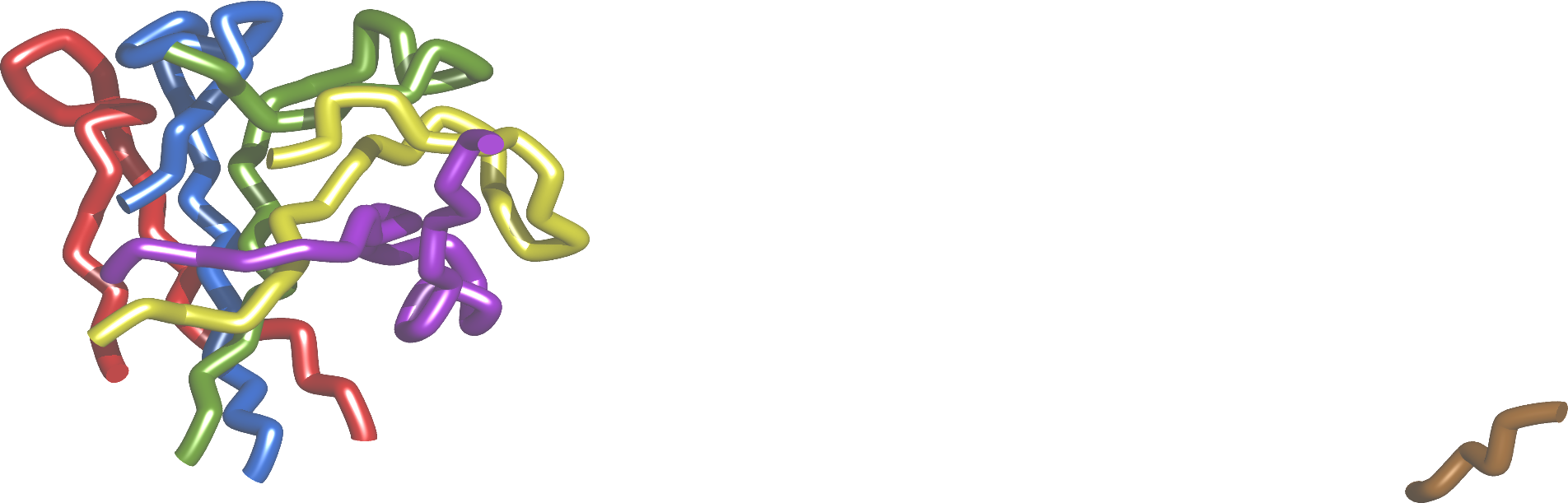

Supplement: Supplementary file 1 [file ijms-23-05247-s001.zip › Suppl. Fig. 3O VLFFFE_cluster_repr_1-3_c3.png]

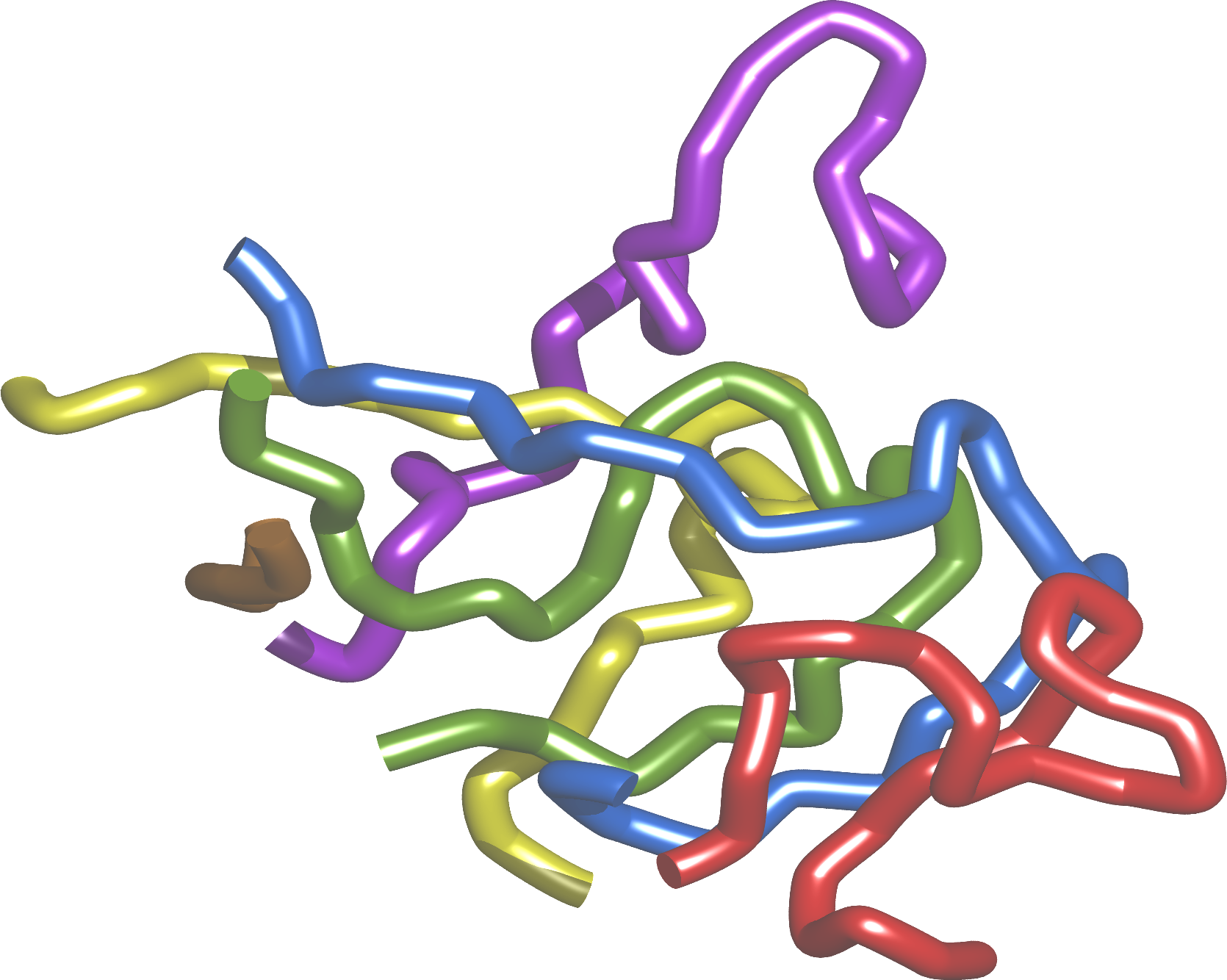

Supplement: Supplementary file 1 [file ijms-23-05247-s001.zip › Suppl. Fig. 3P VVFFWD_cluster_repr_1-3_c0.png]

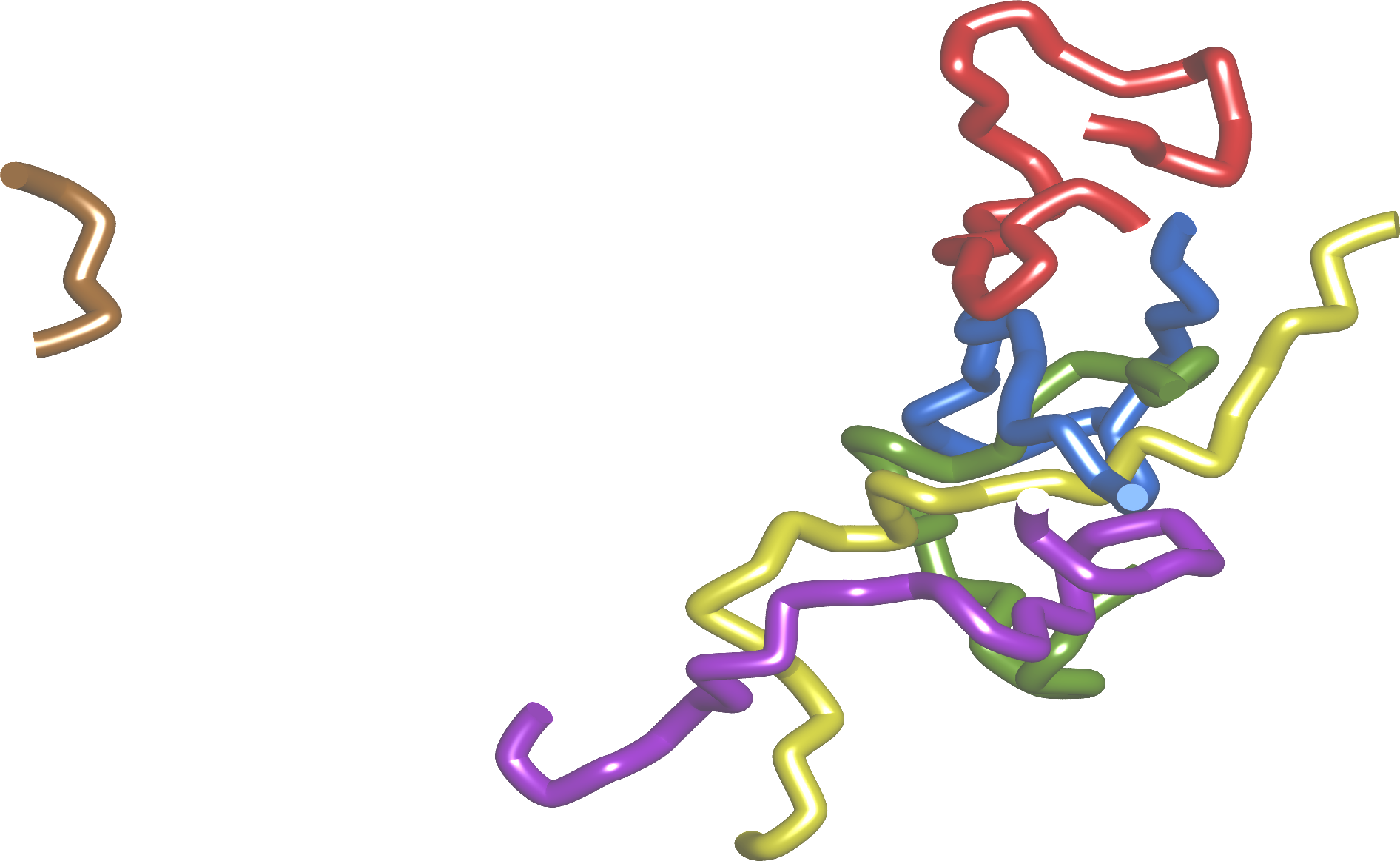

Supplement: Supplementary file 1 [file ijms-23-05247-s001.zip › Suppl. Fig. 3Q VVYFFD_cluster_repr_1-3_c2.png]
